# Geographic variations in place of death and palliative care utilisation in the last three months of life in high-income countries: a systematic review

**DOI:** 10.1186/s12904-025-01869-1

**Published:** 2025-11-21

**Authors:** Nikkita Fewtrell, Halle Johnson, Alex Hughes, Eve Namisango, Mary Abboah-Offei, Kennedy Nkhoma, Emeka Chukwusa

**Affiliations:** 1 King’s College London, Cicely Saunders Institute, Denmark Hill, London, UK; 2 African Palliative Care Association, P.O Box 72518, Kampala, Uganda; 3 School of Health and Social Care, Edinburgh Napier University, Sighthill Court, Sighthill Campus, Edinburgh, UK; 4 Cicely Saunders Institute, King’s College London, Bessemer Road, London, SE5 9PJ UK

**Keywords:** Geographic factors, Healthcare disparities, Health services, End-of-life care, Palliative care, Palliative medicine.

## Abstract

**Background:**

Geographic variations in end-of-life healthcare utilisation may highlight disparities in access to care, including palliative care. Studies demonstrate that place of death and palliative care utilisation are influenced by geographic exposures such as sociodemographic and socioeconomic characteristics, rurality, and health service availability. No systematic review has synthesised the evidence across multiple geographic exposures. This is important to provide conclusions from a broader evidence base and inform equitable commissioning of palliative care services.

**Aim:**

To investigate the influence of geographic exposures on individual-level place of death and palliative care utilisation in the last three months of life, to identify potential disparities in end-of-life healthcare provision.

**Methods:**

In this systematic literature review of quantitative data with narrative synthesis, nine databases were searched for peer-reviewed observational studies published between 1st January 2004 and 1st October 2024. Eligible studies recruited adults in high-income countries and compared two or more geographic exposures. Methodological quality was assessed using the Newcastle-Ottawa Scale.

**Results:**

Of 9,296 studies identified, 51 retrospective studies across 24 countries were included. Rurality was positively associated with home death and decreased palliative care utilisation. Regarding healthcare availability, greater bed availability in hospital or long-term care facilities increased likelihood of death in that facility. Similarly, closer proximity to a hospice or hospital location increased likelihood of death in that facility. Hospital death may be positively associated with residence in certain countries, regions of high population density, and those containing capital cities.

**Conclusions:**

Findings highlight geographic variations as areas of focus for commissioners and policymakers to reduce local end-of-life healthcare inequities. We make recommendations regarding structural care gaps, service efficiency, and innovation in palliative care provision.

**Protocol registration:**

Prospero registration number CRD42019154912.

**Supplementary Information:**

The online version contains supplementary material available at 10.1186/s12904-025-01869-1.

## Background

Healthcare use increases significantly in the last year of life [1, 2], with particular reliance on hospital-based care [1–3]. This dependence intensifies further in the last three months of life [2]. 

Studies use hospital admission rates as a marker of ‘aggressive’ end-of-life care for patients with cancer [4, 5]. This is because admissions are resource-intensive, rarely improve quality of life, and are rarely in line with patients’ preferences [1, 2, 5–7]. In contrast, receipt of specialist palliative care has been associated with improved quality of life [8–11], family satisfaction [12, 13], and psychosocial wellbeing [14] - particularly if initiated early [15–19]. Specialist palliative care facilitates end-of-life care at home or in a home-like environment; [20] the overall most preferred place of death [21–24]. Thus, it seen as an indicator of high-quality care by policymakers [25]. Whilst individual patient preference varies [26], acute or non-specialist care should not be relied upon due to lack of choice [27]. Despite international and national government pledges to provide equitable healthcare for patients with life-limiting illness [28–30], unexplained geographic variations in healthcare utilisation are widely documented [31–34]. Unexplained variations may indicate mismatch between current demand and provision of equitable end-of-life care. Given, the projected increase in serious health suffering globally [35], there is pressing need to examine these variations.

Recent systematic reviews examining access to palliative care by geographic factors using data from Australia, Canada, the Netherlands, the United Kingdom (UK), the United States (US) and New Zealand demonstrated that rural residents had poorer access to hospice care [36–38]. The recent review by Tobin et al. [36] further highlighted that proximity to hospice location was positively associated with receipt of hospice care. No other geographic factors were studied in these reviews, and two of these studies were limited to patients with cancer [37, 38]. 

Other studies use place of death as a proxy measure for healthcare utilisation at end-of-life [39–42]. Whilst place of death can only reflect the final moments of a patient’s healthcare utilisation [43], it is widely accepted as a quality marker given its accessibility via population-level death records and comparability across countries [39, 44, 45]. Place of death is influenced by both environmental and individual factors [46]. A dose-response association was demonstrated between likelihood of death in hospital versus home and increased area deprivation in a systematic review by Davies et al. [47] Greater healthcare availability and social support (e.g. family caregiver input) were associated with home death facilitated by home care in two systematic reviews [48, 49], with Gomes et al. demonstrating that likelihood increased with increasing intensity of home care in the last weeks of life [49]. To our knowledge, few systematic reviews have studied place of death according by geography. One review, focusing on the actual and preferred places of death of rural residents, demonstrated a positive association between rurality and home death in 13 of the 17 studies comparing rural with urban settings [50]. 

The current evidence base explores a limited number of geographic exposures in specific locations, which are not necessarily generalisable. This is challenging for policymakers to interpret in context of their own populations. Our review aims to examine the effect of multiple geographic exposures on two measures of end-of-life healthcare utilisation: place of death and palliative care utilisation. This is important to provide a broader and more diverse evidence base regarding potential gaps in end-of-life healthcare provision, in order to assist authorities in redirecting palliative care resources to where they are needed most [51, 52]. 

## Methods

### Study design

This systematic review was conducted according to Preferred Reporting Items for Systematic Reviews and Meta-Analyses (PRISMA) guidelines [53]. We investigate a subset of outcomes from the protocol of a broader systematic review incorporating other indicators of healthcare utilisation in the last 12 months of life (PROSPERO registration number CRD42019154912) [54]. 

### Eligibility criteria

This review identified observational studies investigating the association between two or more geographic exposures and place of death and/or specialist palliative care utilisation within the last three months of life, for populations aged ≥ 18 years with malignant or non-malignant conditions in high-income countries. We included only adult participants because patterns and preferences for end-of-life care in the paediatric population are known to differ [55]. Geographic exposures (describing either participant residence or place of care/death) included, but were not limited to, those listed in Table 1 below. Socioeconomic geographies were not included in this review, given this was investigated in a recent large systematic review by Davies et al. [47] To avoid measuring funding mechanisms rather than geographic factors, variations related to different healthcare models (for example, a study comparing private versus publicly-funded healthcare systems) were also excluded.

**Table 1:** Examples of geographic exposures included

**Residential or census geographies**: Regional or subregional boundaries (such as states or districts), counties, zip codes, postal areas, national boundaries, output areas (lower, middle and super).
**Urbanisation level or settlement geographies**: Rural (non-metropolitan), urban (metropolitan), peri-urban, population density.
**Proximity to health facilities**: Distance or travel time.
**Service availability**: Counts of health facility within a geographic unit, facility size or bed availability, healthcare worker availability or working hours per capita/per number of population.
**Healthcare boundaries**: Areas serving as healthcare planning regions, which determine local resource allocation decisions to permit or restrict access to services or interventions. For example, Integrated Care Boards (ICBs), Clinical Commissioning Groups (CCGs), Hospital Referral Regions (HRRs), Cancer Networks, other healthcare catchment areas.
**Geopolitical or administrative geography boundaries**: Government Office Regions, electoral wards, districts.

Place of death outcomes included home, hospital, palliative care setting (including inpatient hospice or hospital palliative care bed), or long-term care facility (defined as care homes, nursing homes and other residential care settings for the purposes of this review).

Specialist palliative care outcomes could be provided in any setting. Generalist provision of palliative care (e.g. general practitioner (GP) input) was excluded. Individual-level outcome data were required to analyse results in the context of participants’ sociodemographic and clinical characteristics. [56],^ P.298^ A statistical measure of association or correlation such as odds ratio (OR) with a geographic exposure was required in the analysis of included studies. Descriptive studies presenting results as crude proportions or percentages were excluded, as these would limit ability to make direct comparisons between study outcomes and between quality assessments.

Peer-reviewed prospective or retrospective observational studies in any language were included. Only studies conducted in high-income countries to minimise the influence of confounding differences between healthcare systems of low- and middle-income countries [57]. High-income countries were defined according to the World Bank classification based on Gross National Income per capita thresholds [58]; those included have been classified as high-income for at least 50% of the 20-year time period of this literature search (listed in supplementary text 1).

### Data sources and search strategy

Nine electronic databases were searched without language restriction on 1 st October 2024: MEDLINE, Embase, PsycINFO, CINAHL, Scopus, ProQuest, ASSIA, Web of Science, and HMIC. The full search strategy (Supplementary text 2) was developed in MEDLINE, with medical subject headings (MeSH) and key words linked with AND/OR Boolean operators. This was translated to the syntax of the other databases, with any new subject headings identified incorporated into all other database search strategies as additional keywords for comprehensiveness. Publication date was limited to 1 st January 2004 to 1 st October 2024. This 20-year time period was chosen to avoid capturing older data that no longer reflects the health utilisation norms of a particular location. The use of recent studies also increases the relevance of this review’s findings to current healthcare systems.

To restrict results to high-income countries as defined in this review, country-specific subject headings were applied if available within database syntax.

A filter for observational studies was applied to MEDLINE, Embase and CINAHL searches. This was developed using the Scottish Intercollegiate Guidelines Network (SIGN) filter for observation studies [59], combined with terms related to routine data and registries identified from appendix 1 of a systematic review of similar design [47]. 

### Study selection

Following de-duplication, studies were screened by title and abstract. Full-text reports were then assessed for inclusion according to eligibility criteria. Literature screening was performed using Covidence software, with queries resolved during meetings with supervisors. EndNote 21 software was used for citation management.

### Quality assessment

Quality assessment was performed using the Newcastle-Ottawa Scale (NOS) (Supplementary text 4); a tool with established content validity suitable for non-randomised studies [60]. According to scoring used in previous systematic reviews [61–63], studies were judged to be of high quality if they scored 8 or more points, medium quality if 6 or 7 points, or low quality if under 6 points. The cohort study version of NOS was used to assess quality of retrospective observational and mortality-follow back study designs. Cross-sectional studies were appraised using an adapted version of the case-control NOS [64]. Adaptations were made accordingly to suit the research question, utilising similar domains to other systematic reviews [61, 63, 65, 66]. 

No single confounder was deemed most important to adjust for, given the heterogeneity of variables measured across studies. Instead, this was judged according to each specific study design [65]. Follow up or data availability rate was assessed adequate if either over 90%, or if a lower rate was clearly described or justified. This high threshold was set due to anticipated use of abundant population-level data.

### Data extraction and synthesis

A standard data extraction form (supplementary text 3) was used, with any statistical effect measure between a geographic exposure and outcome recorded.

A narrative synthesis was performed. This is the synthesis method of choice for systematic reviews of similar design [36, 37, 67], where meta-analysis is not feasible due to heterogeneity in variable definitions and measures of association. Given the broad inclusion criteria of this review, narrative synthesis also enabled exploration of study contexts, for example, differences between cancer- and dementia-specific populations. Statistically significant results were mapped out according to geographic subgroups, noting participant characteristics, location and the direction of association, in order to identify patterns in outcome.

## Results

### Study selection

**Fig. 1:**
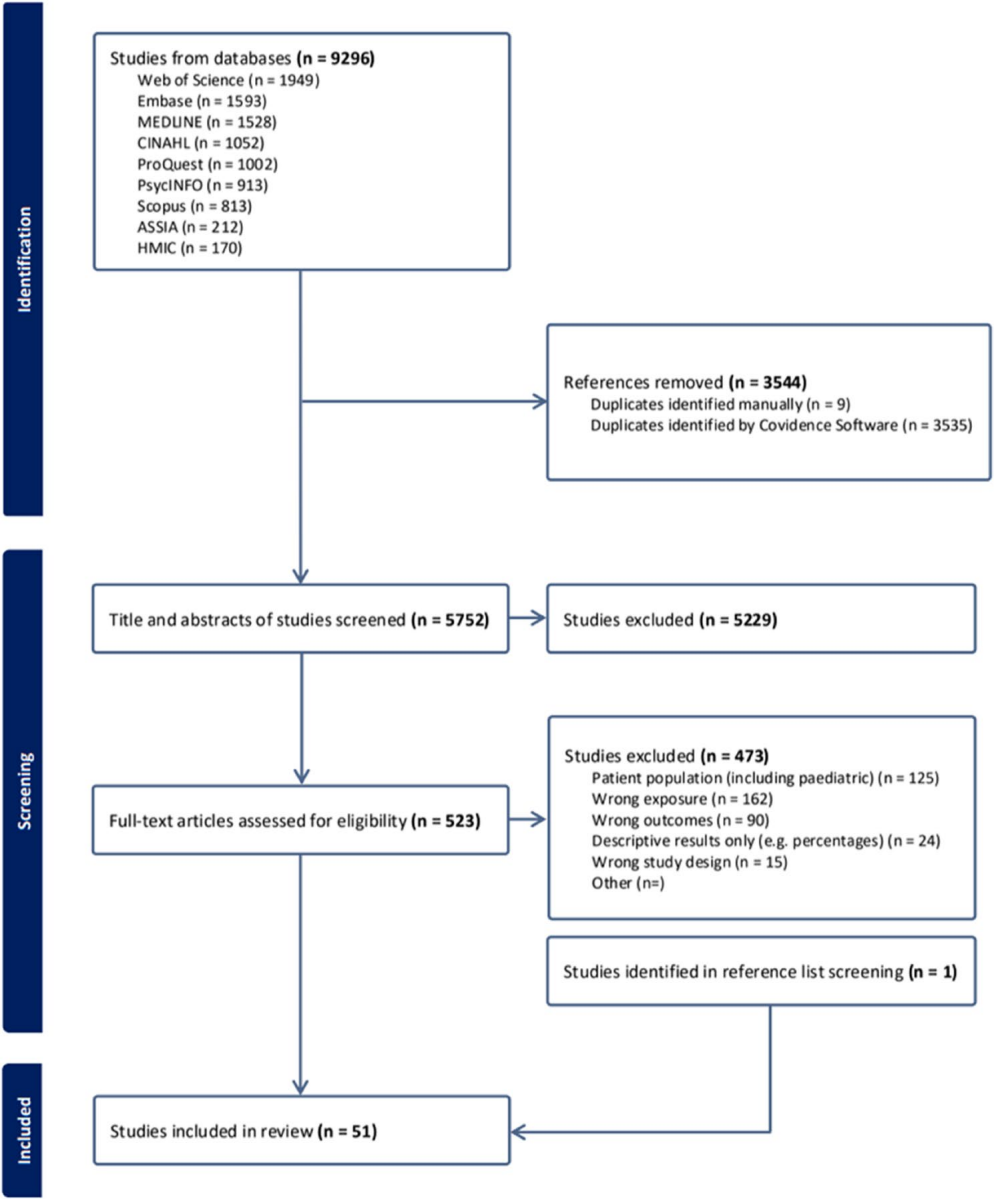
Preferred Reporting Items for Systematic Reviews and Meta-Analyses (PRISMA) flow diagram [53]

This database search yielded 9,296 studies, of which 5,752 studies remained after duplicate removal. Five hundred and twenty-three full-text articles required screening due to the nature of the exposure; geographic exposures were often not mentioned in abstracts and instead only found within study tables. Fifty-one studies met final inclusion criteria. Full selection process is detailed in Fig. 1.

### Study characteristics

The literature search identified *n* = 51 retrospective observational studies: *n* = 38 analysed place of death and *n* = 15 analysed palliative care utilisation. Two studies reported on both outcomes. Several authors investigated multiple geographic exposures of interest.

Over 20,969,942 total participants were represented by studies in *n* = 24 countries: Australia (*n* = 3), Belgium (*n* = 6), Canada (*n* = 7), Chile (*n* = 1), Czech Republic (*n* = 1), Denmark (*n* = 1), England (*n* = 7), Finland (*n* = 1), France (*n* = 2), Hungary (*n* = 1), Ireland (*n* = 1), Italy (*n* = 5), Japan (*n* = 1), the Netherlands (*n* = 6), New Zealand (*n* = 1), Norway (*n* = 1), Spain (*n* = 5), Scotland (*n* = 2), South Korea (*n* = 3), Sweden (*n* = 2), Switzerland (*n* = 1), Taiwan (*n* = 1), United States (*n* = 18), and Wales (*n* = 3). One study required translation from Spanish [68]. 

The majority of study participants were aged over 65 years, with the exception of younger populations in two studies recruiting head and neck cancer patients [69] and cervical cancer patients [70]. The later study was the only study with an all-female population, with the remaining mixed-gender. Marital status was reported in *n* = 20 studies, which ranged from 23.3%[71] to 66.1%[72]. Ethnicity was reported in *n* = 13 studies, all based in the US, with 50.4%[70] to 92.84%[73] of participants reported as white, with the exception of Shepard et al. [74] recruiting a Mexican-American population.

Twenty-six studies recruited disease-specific populations: Cancer in *n* = 21 studies, dementia in four studies and Parkinson’s disease in one study. Of the remaining *n* = 25 studies, *n* = 14 aimed to recruit participants with conditions likely to represent typical healthcare utilisation patterns approaching end of life. This was achieved by either excluding deaths that were sudden or related to external causes, or by limiting inclusion to participants with conditions deemed most likely to benefit from palliative care.

Logistic regression was the most common method of statistical analysis. Most authors reported association using adjusted OR; others chose prevalence ratio, relative risk ratio and regression coefficients.

### Quality assessment results

Results of quality assessment according to the Newcastle-Ottawa Scale (NOS) are shown in Table 2. The questions considered in each category can be found in supplementary text 4.

Most studies were of high or medium quality according to the NOS. Of the cohort and mortality follow-back studies, 69% (18 of 26) were rated high-quality and the remaining 31% (8 of 26) were rated medium-quality. Of the cross-sectional studies, 80% (20 of 25) were rated high-quality, 16% (4 of 25) were rated medium-quality, and 4% (1 of 25) were rated low-quality.

**Table 2:** Newcastle-Ottawa scale quality assessment results

**Cohort/mortality follow back studies**												
**Study ID**	**Selection points**					**Comparabi-lity points**		**Outcome points**				**Total**
	**1**	**2**	**3**	**4**		**1**		**1**	**2**	**3**		
Phillips 2023 [75]	✯	✯	✯	N/A	**3**	✯✯	**2**	✯	✯	✯	**3**	**8**
Penning 2017 [76]	✯	✯	✯	N/A	**3**	✯✯	**2**	✯	✯	✯	**3**	**8**
Cross 2023 [77]	✯	✯	✯	N/A	**3**	✯✯	**2**	✯	✯	**-**	**2**	**7**
Johnson 2005 [78]	**-**	✯	✯	N/A	**2**	✯✯	**2**	✯	✯	✯	**3**	**7**
Kuo 2017 [69]	✯	✯	✯	N/A	**3**	✯✯	**2**	✯	✯	✯	**3**	**8**
Kwak 2008 [79]	**-**	✯	✯	N/A	**2**	✯✯	**2**	✯	✯	✯	**3**	**7**
Morden 2012 [80]	✯	✯	✯	N/A	**3**	✯✯	**2**	✯	✯	✯	**3**	**8**
Maddison 2012 [81]	✯	✯	✯	N/A	**3**	✯✯	**2**	✯	✯	✯	**3**	**8**
Hu 2014 [82]	✯	✯	✯	N/A	**3**	✯✯	**2**	✯	✯	✯	**3**	**8**
Lavergne 2015 [83]	✯	✯	✯	N/A	**3**	✯✯	**2**	✯	✯	✯	**3**	**8**
Gallais Serezal 2016 [84]	✯	✯	✯	N/A	**3**	✯✯	**2**	✯	✯	✯	**3**	**8**
Assareh 2019 [85]	✯	✯	✯	N/A	**3**	✯✯	**2**	✯	✯	✯	**3**	**8**
Assareh 2020 [86]	✯	✯	✯	N/A	**3**	✯✯	**2**	✯	✯	✯	**3**	**8**
Ostan 2023 [87]	✯	✯	✯	N/A	**3**	✯✯	**2**	✯	✯	✯	**3**	**8**
Nayar 2014 [73]	✯	✯	✯	N/A	**3**	✯✯	**2**	✯	✯	✯	**3**	**8**
Watanabe-Galloway 2014 [88]	✯	✯	✯	N/A	**3**	✯✯	**2**	✯	✯	✯	**3**	**8**
de Nooijer 2020 [89]	✯	✯	✯	N/A	**3**	✯✯	**2**	✯	✯	**-**	**2**	**7**
Kenny 2024 [90]	✯	✯	✯	N/A	**3**	✯✯	**2**	✯	✯	✯	**3**	**8**
Beccaro 2007 [91]	✯	✯	**-**	N/A	**2**	✯✯	**2**	✯	✯	**-**	**2**	**6**
Ko 2014 [92]	✯	✯	✯	N/A	**3**	✯✯	**2**	✯	✯	✯	**3**	**8**
Hutchinson 2018 [93]	✯	✯	✯	N/A	**3**	✯✯	**2**	✯	✯	✯	**3**	**8**
Turkman 2019 [94]	✯	✯	✯	N/A	**3**	**-**	**0**	✯	✯	✯	**3**	**6**
Elting 2020 [95]	✯	✯	✯	N/A	**3**	✯✯	**2**	✯	✯	**-**	**2**	**7**
Mohyuddin 2022 [96]	✯	✯	✯	N/A	**3**	✯✯	**2**	✯	✯	✯	**3**	**8**
Shepard 2023 [74]	✯	✯	✯	N/A	**3**	✯✯	**2**	✯	✯	**-**	**2**	**7**
Ramkumar 2023 [97]	✯	✯	✯	N/A	**3**	✯✯	**2**	✯	✯	✯	**3**	**8**
**Cross-sectional studies**												
**Study ID**	**Selection points**					**Comparabi-lity points**		**Outcome points**				**Total**
	**1**	**2**	**3**	**4**		**1**		**1**	**2**	**3**		
Paredes 2019 [68]	✯	✯	✯	✯	**4**	✯✯	**2**	✯✯	✯	**3**		**9**
Reyniers 2015 [71]	✯	✯	**-**	✯	**3**	✯✯	**2**	✯✯	✯	**3**		**8**
Sheu 2019 [70]	✯	✯	✯	✯	**4**	✯✯	**2**	✯✯	✯	**3**		**9**
Forma 2020 [98]	✯	✯	✯	✯	**4**	**-**	**0**	✯✯	✯	**3**		**7**
Menec 2007 [99]	✯	✯	✯	✯	**4**	✯✯	**2**	✯✯	✯	**3**		**9**
Temkin-Greener 2012 [100]	✯	✯	✯	✯	**4**	✯	**1**	✯✯	✯	**3**		**8**
Gao 2014 [46]	✯	✯	✯	✯	**4**	✯✯	**2**	✯✯	✯	**3**		**9**
Cohen 2008 [101]	✯	✯	✯	✯	**4**	✯✯	**2**	✯✯	✯	**3**		**9**
Houttekier 2010 [102]	✯	✯	✯	✯	**4**	✯✯	**2**	✯✯	✯	**3**		**9**
Houttekier 2010 [103]	✯	✯	✯	✯	**4**	✯✯	**2**	✯✯	✯	**3**		**9**
Luta 2016 [104]	✯	✯	✯	✯	**4**	✯✯	**2**	✯✯	✯	**3**		**9**
Cheon 2023 [105]	✯	**-**	**-**	✯	**2**	✯✯	**2**	✯	✯	**2**		**6**
Klinkenberg 2005 [106]	✯	**-**	**-**	✯	**2**	✯✯	**2**	✯	✯	**2**		**6**
Lackan 2009 [107]	✯	✯	✯	✯	**4**	✯✯	**2**	✯✯	✯	**3**		**9**
Neergaard 2012 [108]	✯	✯	✯	✯	**4**	✯✯	**2**	✯✯	✯	**3**		**9**
Alonso-Babarro 2013 [72]	✯	✯	✯	✯	**4**	✯✯	**2**	✯✯	✯	**3**		**9**
Ohlen 2017 [109]	✯	✯	✯	✯	**4**	✯✯	**2**	✯✯	✯	**3**		**9**
Lee 2018 [110]	✯	**-**	**-**	**-**	**1**	✯✯	**2**	✯	✯	**2**		**5**
Lopez-Valcarcel 2019 [111]	✯	✯	✯	✯	**4**	✯✯	**2**	✯✯	✯	**3**		**9**
Chukwusa 2019 [51]	✯	✯	✯	✯	**4**	✯✯	**2**	✯✯	✯	**3**		**9**
May 2020 [112]	✯	✯	**-**	**-**	**2**	✯✯	**2**	✯	✯	**2**		**6**
Orth 2021 [113]	✯	✯	**-**	✯	**3**	✯✯	**2**	✯✯	✯	**3**		**8**
Abe 2022 [114]	✯	✯	✯	✯	**4**	✯✯	**2**	✯✯	✯	**3**		**9**
Chukwusa 2020 [52]	✯	✯	✯	✯	**4**	✯✯	**2**	✯✯	✯	**3**		**9**
Xu 2020 [115]	✯	✯	**-**	✯	**3**	✯✯	**2**	✯✯	✯	**3**		**8**

N/A - Not applicable

### Narrative synthesis

Results of individual studies are shown in supplementary Tables 3 and 4. We synthesise statistically significant results by subgroup of geographic exposure: Rural-urban variations (*n* = 24 studies); country (*n* = 5 studies); region (*n* = 13 studies); distance or drive time to a healthcare facility (*n* = 6 studies); and health service availability, which includes both facility capacity (*n* = 10 studies) and healthcare worker availability (*n* = 4 studies).

#### Rural-urban variation: place of death

Rural residence was positively associated with death at home in four of five studies comparing this to either hospital or other places of death [68, 103, 105, 109, 110]. In the study demonstrating a negative association, authors combined home and hospice death in their analysis, which is likely to have influenced results [69]. There were mixed findings regarding the association between rurality and likelihood of death in a hospital, long-term care facility or hospice.

Regarding rural-urban variation and hospital death compared to other places of death: Nine studies reported increased likelihood in urban areas [68, 71, 77, 83, 98, 103, 109, 110, 113] (with two studies demonstrating this trend across multiple countries [71, 103]) and eight studies reported increased likelihood in rural areas [69, 70, 81, 82, 86, 99, 100, 112]. One study was equivocal [76]. No studies demonstrated a dose-response relationship. One study’s findings were only significant in those with severe dementia [113]. Strength of association was unclear in two studies due to choice of geographic classification: Assareh et al. [86] found greater likelihood of hospital death in ‘outer regional’ and ‘remote’, but not ‘inner regional’ or ‘very remote’ areas in Australia. Hu et al. [82] categorised results according to Canadian region (the north, central and south regions labelled ‘mixture of suburban/rural/remote’ had higher odds of hospital death compared to Edmonton and Calgary labelled ‘urban/suburban’).

Regarding the association between of rurality and death in a long-term care facility compared to hospital death: Two found increased likelihood for rural residents [98, 103] and two found increased likelihood for urban residents [99, 112]. Only one study compared long-term care facility death to home death [109]. 

The three studies analysing hospice death according to rurality were not comparable due to heterogenous outcomes [69, 70, 84] (e.g. Gallais Serezal et al. [84] classified death in hospital ‘palliative care support beds’ as hospice deaths).

#### Rural-urban variation: palliative care utilisation

Urban residence was positively associated with receipt of palliative care in the four studies comparing this to no receipt of palliative care [73, 90, 97, 113], although one study (differentiating ‘hospice’ and ‘palliative care’) found this association only for hospice care three or more days before death [97]. The remaining studies captured a variety of durations of palliative care. No dose-response relationship was demonstrated by the two studies comparing levels of increasing rurality [73, 97], although it is noted two studies found a dose-relationship present among those who had ‘ever’ used hospice [73, 88]. One study’s findings were only significant in patients with moderate severity of dementia [113]. 

In the three studies comparing timing or intensity of palliative care utilisation, there was no consistent association. Two found rural residence associated with receiving palliative care for ≥ 90 days (without dose-response relationship). However, authors’ results were not comparable due to differences in outcome definition (e.g. one investigating timing among only hospice users [93], the other investigating timing among both hospice and non-hospice users) [97]. 

#### Cross-national variation: place of death

Three studies analysed place of death by country: Houttekier et al. [102] studied five countries; Cohen et al. [101] studied six countries; and Reyniers et al. [71] studied eleven countries. Two of these studies analysed death in populations with dementia [71, 102], with the third studying all causes of death. [101]

England, the Netherlands, Belgium and Wales were the only countries to be involved in all three studies on place of death, therefore demonstrated the most consistent associations. From these results, odds of hospital death versus home death were higher in England compared to the Netherlands and Belgium in all three studies. Residence in Scotland was also positively associated with hospital death in two studies [101, 102]. 

Home death versus hospital death was strongly associated with residence in the Netherlands in two studies [71, 102] (with odds of dying at home in the Netherlands was more than 15 times (OR 15.43, 95% CI 12.42–19.17) the odds for those resident in England, according to Houttekier et al. [102]).

Similarly, death in a long-term care facility (versus hospital) was most likely in the Netherlands and Belgium, according to both studies investigating nursing home as a place death [71, 102]. This was 38 times more likely (OR 38.0, 95% CI 31.2–46.1) in the Netherlands compared to France, according to Reyniers et al. [71] Long-term care facility death was also more likely compared to home death in the Netherlands and Wales in both studies analysing this outcome [71, 102]. 

No studies measured the association between country and hospice death.

#### Cross-national variation: palliative care utilisation

No consistent association was found between country of residence and receipt of specialist palliative care across the two studies included [89, 92]. 

#### Distance and drive time: place of death

Proximity to a hospice or hospital facility was generally associated with higher likelihood of dying in that facility.

Three of four studies reported increased odds of hospital death with a shorter distance or faster drive time to hospital locations (from home [51, 107] or from nursing home [100]). One study found this only significant for rural residents and for those residing less than 50 min from hospital [51]. On the contrary, Assareh et al. [86] found hospital death associated with further distance from hospital locations, however the association did not persist in a separate stratified analysis of palliative care patients only.

Regarding hospice death, two studies in the UK showed increased likelihood of hospice death compared to home death with shorter drive time to hospice locations [51, 52]. One demonstrated a dose-response association across several UK Government Office Regions [52]. 

#### Distance and drive time: palliative care utilisation

No studies analysed the association between palliative care utilisation and distance or drive time to healthcare facilities.

#### Regional variation: place of death

No single region was represented by more than one study comparing the same outcomes.

A possible association was observed between hospital death and residence in regions with a high population density or where the capital city is located. This was found in in six of seven studies investigating hospital deaths [46, 69, 70, 75, 104, 109, 111]: The Stockholm healthcare region in Sweden [109], the London UK health authority region [46], the northern region of Taiwan containing Taipai [69], the Northeast region of the US [70, 75] and the autonomous community of Madrid in Spain [111]. (The latter study of cancer deaths in Spain also demonstrated this association for province capitals and large municipalities [111]). 

There was also possible association between residence in less-densely populated regions and greater likelihood of home death, according to three of four studies [46, 78, 106, 111]: West and Midwest of the US [78], Northeast of the Netherlands [106], and South West region of the UK [46]. 

The studies analysing long-term care facility deaths [46, 78, 104, 109, 111] and hospice deaths [70, 78] according to region did not demonstrate any observable trend.

#### Regional variation: palliative care utilisation

No consistent association was demonstrated between region of residence and palliative care utilisation in three studies. Two analysed Italian regional disparities in home palliative care provision, however produced incomparable results [74, 87, 91]. 

#### Health service availability: place of death

Greater availability of healthcare facility beds increased likelihood of dying in certain facilities. Five of six studies reported greater availability of hospital bed as a predictor of hospital deaths [71, 80, 102, 103, 107, 113]. Hospital death was also associated with other measures of care availability: fewer long-term care beds in three studies [71, 104, 115], a decreased supply of GPs in one study [71], and the absence of a palliative care home team in another study [72]. Healthcare availability in New Zealand was an exception to these trends in one of these studies [71]. 

Death in a long-term care facility was more likely with greater long-term care bed availability in all four studies investigating this variable (compared to home and/or hospital) [102–104, 115]. 

Home death was more likely with fewer long-term care facility beds in two studies [102, 115] and fewer hospital beds in two studies [102, 114]. 

Only one study analysed place of death according to palliative care availability [72]. 

#### Health service availability: palliative care utilisation

Conclusions could not be drawn from the three studies investigating healthcare availability factors due to lack of common independent variable [80, 94, 113]. Two found increased likelihood of receiving hospice care with reduced provision of other forms of end-of-life care (fewer registered nurse staffing hours [113] and the lack of hospital palliative care beds [94]), suggesting non-specialist palliative care is being provided in its place [113]. However, the latter study results were unadjusted.

## Discussion

### Main findings

We found evidence of geographic variation in place of death and palliative care utilisation in the final three months of life. Despite the inevitable influence of unmeasured physical and sociocultural characteristics of each study’s resident population [87, 104, 111] the following consistent associations were demonstrated in this review.

Receipt of specialist palliative care was positively associated with urban residence. Home death was positively associated with rural residence.

Greater bed availability in hospitals and long-term care facilities increased likelihood of death in that facility. Similarly, closer proximity or drive time to a hospice or hospital location increased likelihood of death in that facility.

Regarding cross-national variation in place of death, England and Wales were among those with highest likelihood of hospital death in comparison to the Netherlands and Belgium. The Netherlands also demonstrated highest odds of an out-of-hospital death among included studies. These findings were extracted from the few cross-national comparison studies meeting inclusion criteria for their comparable methodologies (clearly-defined adult populations and exclusion of solely descriptive data). However, we make these comparisons with caution due to between-country differences in national healthcare models, cultural expectations and data collection methods.

### What this study adds

Our findings confirm that rural residents are less likely to receive specialist palliative care (as demonstrated in previous systematic reviews [36–38, 116]). This aligns with our findings that greater distance or drive time from a hospice facility, often located in urban areas, is associated with decreased likelihood of death in a hospice facility. Interpreted together with our findings that rural residents are more likely to die at home (updating the evidence base with the addition of four studies published between 2017 and 2023[68, 105, 109, 110]), this may reflect rural residents dying at home without adequate support [68, 105, 110] (although unmeasured non-specialist community end-of-life care cannot be accounted for) [51]. 

Furthermore, it is possible that individuals with greater palliative care access also have reduced likelihood of acute hospital death, as demonstrated by two included studies not pooled in this review due to different exposure variables (proximity to a palliative care programme [83] and availability of a palliative care home team [72]). This would reflect conclusions from a previous literature review demonstrating the presence of palliative care in any setting increased the likelihood of death in that setting [48]. Whilst one might therefore expect urban residents with greater access to specialist palliative care to have a lower likelihood of hospital death, our findings regarding author-defined rural-urban variation and hospital death were equivocal. We did observe a possible association between hospital death and regions known to be population-dense or contain capital cities in our regional variation analysis, however these study authors did not themselves define these regions as ‘urban’. Further research is therefore required into urban-rural variation and hospital death.

Several possible contextual factors may play a role in the heterogeneity of our findings on the association between urbanisation level and hospital death. For ease of comparability, we summarise the key characteristics of included studies reporting on rural-urban variation and hospital death in supplementary Table 5. Additional unmeasured contextual factors may have also influenced our findings. For example, regarding physical infrastructure, three of the eight studies demonstrating an association between hospital death and rural residence were conducted in Canada [81, 82, 99]. Canadian rural areas may have particularly challenging terrain [83], thus a hospital death may reflect a patient who is prevented from travelling home at the end of life [83, 86]. Conversely, for those studies associating hospital death with urban residence, dense built environments (such as multistorey apartment blocks) [117] may make the receipt of specialist palliative home care, which would facilitate an out-of-hospital death, less feasible in some cities.

Healthcare structural and funding differences may also play a role. For example, a city’s residents may benefit from high-quality palliative care integrated into its city hospital services, thus making hospital death most appropriate [118]. This is likely to be exacerbated if healthcare planning region boundaries fund uniformly urbanised or uniformly rural areas.

Finally, sociocultural factors, alongside urbanization levels, may also contribute to heterogeneity of findings. These include cultures of ‘hospital-centredness’ in certain settings [111], and social fragmentation (such as fewer cohabiting relatives able to provide home care) [49, 117], with social support a known determinant of place of death [49]. 

We also demonstrate that healthcare availability is likely to contribute to inequitable end-of-life care provision, in line with previous studies [48, 49, 119]. Regarding bed availability, it is noted that five of the six included studies demonstrating an increased likelihood of death in a long-term care or hospital with increasing bed availability recruited populations of older adults [71, 102–104, 107], with two specific to dementia populations [71, 102]. Patients with dementia are projected to have the highest proportional increase of serious health-related suffering by 2060 [35]. Given this patient group are particularly likely to benefit from a stable end-of-life environment such as an long-term care facility (avoiding hospital environments which may exacerbate agitation and disorientation) [120], filling this gap in out-of-hospital bed provision will become increasingly urgent.

Cross-national variations in place of death identified in this review may reflect inequitable health policy, as suggested in a multinational 2008 all-age analysis comparing home versus hospital death, which found differences that were only partially explained by sociodemographic factors, cause of death and bed availability [121]. However, we make conclusions with caution given the paucity of eligible studies in this review. Of note, many cross-national comparison studies did not meet inclusion criteria because authors were only willing to provide descriptive results (such as percentages), likely due to between-country health system differences. This includes the roles and expectations of healthcare professionals. For example, we note that the two included studies analysing receipt of palliative care by country (not pooled in this review due to different countries analysed) demonstrated that countries with lower odds of specialist palliative care use had greater odds of non-specialist, GP-led palliative care [89, 92]. Similarly, Ko et al. [92] acknowledged the high availability of specialised nursing home beds supported by elderly care physicians in the Netherlands (as opposed to primary care input alone) by excluding the Netherlands from their analysis. Other factors to consider in cross-national comparisons include variations in data storage, collection and terminology, which led to the exclusion of Hungary and New Zealand from cross-national comparisons in Reyniers et al.’s study [71]. 

Recommendations for local commissioners and policymakers to address end-of-life care inequities are summarised in Table 3. These are made according to the Donabedian model for assessing quality of a health care system: Structure (its attributes), process (its activity to deliver care) and outcome (its effect on patients) [122]. 

As per the main findings in this review, our structural recommendations focus on actions to strengthen access to specialist palliative care, particularly community-based care, for those in rural locations and those at furthest proximity from facilities. Whilst many structural interventions may be resource- and time-costly, we highlight the importance of first empowering patients to make informed decision-making surrounding the care options already available in our process recommendations. This is because preferences may be influenced by what the patient believes is feasible in their circumstances, rather than their genuine wishes [41, 123]. For example, if a patient believes home visits would be too infrequent to meet their symptom control needs, they may feel the only safe alternative is to be cared for in hospital [21, 124].

Regarding outcomes, many authors of the studies in this review stated that it was the first study of its kind in that specific location or population[71, 82, 85, 89, 102]. Regular needs assessments - tailored to each locality - are required. This reflects the concept of ‘place-based planning’[125], which acknowledges the complex interplay of social determinants that are unique to each area. Extrapolation of readily available data from one locality to another, even if from neighbouring or similar locations, may lead to availability bias[126]. 

More broadly, prospective studies which better adjust for individual-level characteristics, and thus clarify reasons for gaps in healthcare provision, are required. This may also enable identification of individuals’ preferences for healthcare utilisation and places of death[69], as well as the quality, experiences and meaning attached to each setting, not simply their spatial distribution, as emphasised by Gatrell et al. [127],^ P.35^

**Table 3:** Recommendations to reduce end-of-life healthcare inequities

Recommendations to reduce end-of-life healthcare inequities
**Structure**: • Improve integration of palliative care within nursing homes [128] • Use eHealth* to close gaps of physical distance [129] • Strengthen community-based support, for example by providing informal caregivers and long-term care facility staff with training as well as emotional and psychological support [97, 123]. • Ensure new healthcare planning boundaries are not coterminous with known geographic disparities. For example, by ensuring they span across settings with diverse levels of urbanisation and population density [132]. • Expand catchment areas covered by palliative care home teams. • Increase bed numbers at existing hospice or long-term care facilities. • Carry out equity impact assessments for any new or re-developed facilities, anticipating some users may be geographically disadvantaged.
**Process**: • Implement local and national initiatives for advanced care planning [133]. • Offer opportunistic, culturally-sensitive patient education to facilitate patient choice [74, 133, 134] (provided high-quality care options are available [135]). • Identify inefficiencies in the hospital discharge process for dying patients [136] • Improve information-sharing between healthcare agencies to facilitate transitions between places of care towards end of life [117, 134].
**Outcome**: • Perform regular needs assessments specific to each locality. • Future prospective studies which adjust more thoroughly for potential confounding factors (such as local healthcare availability and sociocultural factors) • Future prospective studies which analyse geographic variations alongside patient preference and rationale for choice of healthcare utilisation at end of life.

*eHealth uses information technologies to support healthcare delivery, recognised by the World Health Organization as an increasingly important means of responding to need [130]. In a small integrative review of palliative care patients’ experiences (n=397) [131], eHealth demonstrated promising patient usability and satisfaction

### Strengths and weaknesses of this study

The main strength of this systematic review is its broad eligibility criteria enabling the investigation of multiple geographic exposures according to a combination of two metrics for end-of-life healthcare utilisation, thus increasing generalisability of findings. The search conducted across nine databases with no language restriction identified studies with mostly low to medium risk of bias.

This study has several limitations. Firstly, despite quality assessment results, included studies inherently lack evidence for causation due to their retrospective observational design[137]. Of note, certain patterns of healthcare utilisation or place of death may result from or be determined by environmental exposures, thus there is risk of reverse causation bias[37, 138].

Secondly, certain associations identified in this review have reduced completeness and generalisability due to paucity of studies [139]. For example, no studies reported hospice death outcomes according to country, region or healthcare availability, perhaps in part due to the absence of hospice as a place of death category in some countries’ routine data collection [71, 83, 99, 109]. 

Comparability between studies was further limited by heterogeneity in both exposure (particularly urban-rural classifications) and outcome variable definitions (see supplementary Table 5). This has been a reported challenge in similar systematic reviews [36]. Heterogeneity in participant causes of death across studies may have contributed to the equivocal findings of rural-urban variation on hospital death in this review.

Thirdly, no studies adjusted for use of community-based healthcare services, including home care, despite this being a major factor influencing place of death [48, 49]. Only 25% (13/51) of included studies adjusted for any other form of health service availability, despite authors acknowledging their importance (for example, one study comparing two areas with and without palliative home care teams noted one area had more acute hospital beds [72]). Some studies that did adjust for health service availability confirmed its confounding effect in their models. For example, Houttekier et al. [102] found notably higher odds of nursing home death compared to hospital death in Belgium after adjusting for nursing home bed availability. Kwak et al. [79] found that hospital death was no longer significant after adjustment for hospice use.

Fourthly, regarding statistical analysis, many studies used OR to measure association, which may overestimate common outcomes, such as place of death, compared to proportion or prevalence ratios [108, 140]. 

Finally, our objective outcome variables of place of death and palliative care utilisation in the last three months of life fail to capture other experiences of healthcare utilisation at end of life [115], including non-specialist high-quality palliative care, and indeed the voice of the patients themselves [42]. Other quantitative and qualitative metrics of all forms of healthcare utilisation should be explored in future systematic reviews.

## Conclusion

End-of-life care systems have the power to reinforce inequities unless they are distributed equitably. This systematic review conducted across twenty-four high-income countries has identified variations in place of death according to multiple geographic exposures, and variations in palliative care utilisation according to rurality. These variations may reflect gaps in end-of-life healthcare provision, thus should be areas of focus for healthcare commissioners and policymakers. The structures, processes and outcomes required to address these inequities are likely to require innovative and population-specific approaches.

## Supplementary Information

Supplementary Material 1. Text 1 - Countries meeting inclusion criteria. Text 2 - Search strategy. Text 3 - Standard data collection form. Text 4 - Newcastle Ottawa Quality Assessment Scale (NOS). Table 3 - Results of individual studies: Place of death. Table 4 - Results of individual studies: Specialist palliative care in the last 3 months of life. Table 5 – Key characteristics of studies reporting on rural-urban variation and hospital death. (significant results only).

## Data Availability

All data generated or analysed during this study are included in this published article and its supplementary information files.
