# Supplementary material for: Geographic variations in place of death and palliative care utilisation in the last three months of life in high-income countries: a systematic review
Source: BMC Palliat Care. 2025 Nov 21;24:293. doi: 10.1186/s12904-025-01869-1 (PMC12639923; doi:10.1186/s12904-025-01869-1)
Supplement: Supplementary file 1 — Supplementary Material 1. Text 1 - Countries meeting inclusion criteria. Text 2 - Search strategy. Text 3 - Standard data collection form. Text 4 - Newcastle Ottawa Quality Assessment Scale (NOS). Table 3 - Results of individual studies: Place of death. Table 4 - Results of individual studies: Specialist palliative care in the last 3 months of life. Table 5 – Key characteristics of studies reporting on rural-urban variation and hospital death. (significant results only). [file 12904_2025_1869_MOESM1_ESM.docx]

**Supplementary Materials**

### **Contents**

### Text 1 - Countries meeting inclusion criteria

### Text 2 - Search strategy

### Text 3 - Standard data collection form

### Text 4 - Newcastle Ottawa Quality Assessment Scale (NOS)

### Table 3 - Results of individual studies: Place of death

### Table 4 - Results of individual studies: Specialist palliative care in the last 3 months of life Table 5 – Key characteristics of studies reporting on rural-urban variation and hospital death (significant results only)

### **Text 1: Countries meeting inclusion criteria**

Countries classified as ‘high-income’ by the World Bank for 50% or more of the time period between 1 January 2004 and 29^th^ April 2024.^58^

Aruba, Curacao, Sint Maarten, Saint Martin^a^, Canada, United States, Chile, Uruguay, Brunei, Bahrain, Israel, Kuwait, Oman, Qatar, Saudi Arabia, United Arab Emirates, Hong Kong, Macau/Macao, Republic Of Korea, Andorra, Austria, Belgium, Estonia, Latvia, Lithuania, Croatia, Czech Republic, Hungary, Poland, Slovakia, Slovenia, France, Germany, Gibraltar, United Kingdom (including Channel Islands), Isle of Man^a^, Cayman Islands^a^, Greece, Ireland, Italy, Liechtenstein, Luxembourg, Monaco, Netherlands, Portugal, San Marino, Denmark, Finland, Iceland, Norway, Sweden, Faroe Islands^a^, Spain, Switzerland, Vatican City, Bermuda, Australia, Greenland, Seychelles, Japan, Cyprus, Malta, New Caledonia, Guam, New Zealand, Hawaii, Taiwan, Antigua And Barbuda, The Bahamas, Barbados, Puerto Rico, Saint Kitts And Nevis, Trinidad And Tobago, Singapore, United States Virgin Islands, British Virgin Islands, French Polynesia^b^, Northern Mariana Islands^b^, American Samoa^b^, Turks And Caicos Islands^b^.

1. Subheading available in Embase only.
2. No subheading for this country available on Medline or Embase. (French Polynesia, Northern Mariana Islands and American Samoa assumed to be included under ‘Pacific Islands’ subheading in Medline).

### **Text 2: Search Strategy**

| **MEDLINE**   1. Exp Geography/ 2. Exp Population density/ 3. Exp Population/ 4. Exp Catchment Area, Health/ 5. Exp Neighborhood Characteristics/ 6. exp Health Services/ 7. exp Health Facilities/ 8. exp Health Services Accessibility/ 9. "Health Services Needs and Demand"/ 10. (Geograph* or topograph* or geomedicine or nosograph*).tw 11. (Rural* or urban* or remote or peri-urban or suburb* or metropolitan or non-metropolitan) adj3 (location or area)).tw 12. (City or cities or town* or village* or suburb* or county or country or countryside* or region* or provinc* or district* or state* or council* or border* or boundar* or "output area*" or constituenc* or parish* or postcode or “zip code” or distance or proximity or spatial or contextual or state-level or spatio-temporal or country-level or spatial-trend or socio-spatial or area-level or neighbo?rhood-level or location* or site* or density or nation* or sub-nation* or settlement or small-area or catchment or intra-state or inter-region*).tw 13. (CCG* or “clinical commissioning group*” or “local commissioning group*” or ICB* or “integrated care board*” or "hospital referral region*" or “integration authorit*” or “local health board*” or “sustainability and transformation plan*” or network* or alliance* or board* or trust* or authorit*).tw 14. 1 or 2 or 3 or 4 or 5 or 6 or 7 or 8 or 9 or 10 or 11 or 12 or 13 15. (difference* or variation* or disparit* or vary or varies or distribution* or inequit* or inequalit*).tw 16. exp Healthcare Disparities/ 17. 15 or 16 18. exp Palliative Care/ 19. exp Palliative Medicine/ 20. “Hospice and Palliative Care Nursing”/ 21. Terminal Care/ 22. Hospice Care/ 23. Duration of Therapy/ 24. Episode of Care/ 25. House Calls/ 26. Home Nursing/ 27. ((Hospice or palliat*) adj3 (care or nursing or therapy or utili?ation or "use" or usage or uptake or access* or delivery or coverage or contact or visit* or consult* or enrol?ment or admission or stay or "bed days" or "bed occupancy" or proximity or distance)).tw. 28. ((Length or duration) adj3 (Hospice or palliat*) adj3 (utili?ation or "use" or usage or uptake or access* or delivery or coverage or contact or visit* or enrol?ment or admission or stay)).tw. 29. (“Specialist palliative*” or “in?patient palliative*” or “out?patient palliative*” or “hospital palliative*” or “community palliative*” or “home palliative*”).tw 30. (Place adj3 care) OR (Place adj3 death) 31. 18 or 19 or 20 or 21 or 22 or 23 or 24 or 25 or 26 or 27 or 28 or 29 or 30 32. exp Terminally Ill/ 33. (palliative or terminal* or EOL or EOLC or "end of life*" or "end?of?life*" or “end of their lives” or dying or “supportive care” or “symptomatic treatment” or “irreversible condition” or “terminal condition” or fatal illness).tw 34. ((incurable* or life?limit* or advanc* or progressiv* or agressiv* or end) adj2 (diagnos* or diseas* OR illnes* or cancer* or malignan* or non-malignan* or stage* or dementia* or failure* or heart*)). tw 35. ((end or last or final) adj3 life). Tw 36. (expected adj3 die).tw 37. (imminent adj3 death).tw. 38. (“Dying soon” or “expected death” or “imminently dying” or moribund).tw 39. 32 or 33 or 34 or 35 or 36 or 37 or 38 40. Exp aruba/ or exp curacao/ or exp sint maarten/ or exp canada/ or exp united states/ or exp chile/ or exp uruguay/ or exp brunei/ or exp bahrain/ or exp israel/ or exp kuwait/ or exp oman/ or exp qatar/ or exp saudi arabia/ or exp united arab emirates/ or exp hong kong/ or exp macau/ or exp "republic of korea"/ or exp andorra/ or exp austria/ or exp belgium/ or exp estonia/ or exp latvia/ or exp lithuania/ or exp croatia/ or exp czech republic/ or exp hungary/ or exp poland/ or exp slovakia/ or exp slovenia/ or exp france/ or exp germany/ or exp gibraltar/ or exp united kingdom/ or exp greece/ or exp ireland/ or exp italy/ or exp liechtenstein/ or exp luxembourg/ or exp monaco/ or exp netherlands/ or exp portugal/ or exp san marino/ or exp "scandinavian and nordic countries"/ or exp spain/ or exp switzerland/ or exp vatican city/ or exp bermuda/ or exp australia/ or exp greenland/ or exp seychelles/ or exp japan/ or exp cyprus/ or exp malta/ or exp new caledonia/ or exp guam/ or exp new zealand/ or exp hawaii/ or exp taiwan/ or exp "antigua and barbuda"/ or exp bahamas/ or exp barbados/ or exp puerto rico/ or exp "saint kitts and nevis"/ or exp "trinidad and tobago"/ or exp united states virgin islands/ or exp singapore or exp british virgin islands/ or exp pacific islands/ 41. 14 and 17 and 31 and 39 and 40 42. limit 41 to humans 43. limit 42 to yr="2004 -Current" 44. limit 43 to "all adult (19 plus years)” 45. Epidemiologic studies/ 46. Exp case control studies/ 47. Exp cohort studies/ 48. Cross-sectional studies/ 49. Exp Registries/ 50. (epidemiolog*or case control or cross?section* or cross section* or cohort* or Cohort analy$ or survey* or observational* or retrospectiv* or prospective* or longitudinal or routine?data or routine data or population?based or population based).tw 51. (cohort or follow up or observational) adj (study or studies).tw. 52. (registr*) adj2 (cancer or death or disease or national or central or regional).tw 53. (Population*) adj2 (study* or sample* or data* or design* or based).tw 54. linked adj3 data.tw 55. administrative adj2 **(**data or record*).tw 56. or/45-55 57. 44 and 56 |
| --- |
| **EMBASE**   1. exp geography/ 2. population/ 3. urban rural difference/ 4. exp rural area/ 5. rural population/ 6. rural hospital/ 7. rural health care/ 8. exp urban area/ 9. urban population/ 10. urban health/ 11. urban hospital/ 12. suburban population/ 13. exp “catchment area (health)”/ 14. neighborhood characteristic/ 15. health care availability/ 16. (Geograph* or topograph* or geomedicine or nosograph*).tw 17. ((Rural* or urban* or remote or peri-urban or suburb* or metropolitan or non-metropolitan) adj3 (location or area)).tw 18. (City or cities or town* or village* or suburb* or county or country or countryside* or region* or provinc* or district* or state* or council* or border* or boundar* or "output area*" or constituenc* or parish* or postcode or “zip code” or distance or proximity or spatial or contextual or state-level or spatio-temporal or country-level or spatial-trend or socio-spatial or area-level or neighbo?rhood-level or location* or site* or density or nation* or sub-nation* or settlement or small-area or catchment or intra-state or inter-region*).mp 19. (CCG* or “clinical commissioning group*” or “local commissioning group*” or ICB* or “integrated care board*” or "hospital referral region*" or “integration authorit*” or “local health board*” or “sustainability and transformation plan*” or network* or alliance* or board* or trust* or authorit*).tw 20. 1 or 2 or 3 or 4 or 5 or 6 or 7 or 8 or 9 or 10 or 11 or 12 or 13 or 14 or 15 or 16 or 17 or 18 or 19 or 20 21. (difference* or variation* or disparit* or vary or varies or distribution* or inequit* or inequalit*).tw 22. health care disparity/ 23. 21 or 22 24. exp *palliative therapy/ 25. exp *palliative nursing/ 26. *terminal care/ 27. *hospice care/ 28. ((Hospice or palliat*) adj3 (care or nursing or therapy or utili?ation or "use" or usage or uptake or access* or delivery or coverage or contact or visit* or consult* or enrol?ment or admission or stay or "bed days" or "bed occupancy" or proximity or distance)).tw. 29. ((Length or duration) adj3 (Hospice or palliat*) adj3 (utili?ation or "use" or usage or uptake or access* or delivery or coverage or contact or visit* or enrol?ment or admission or stay)).tw. 30. ("Specialist palliative*" or "in?patient palliative*" or "out?patient palliative*" or "hospital palliative*" or "community palliative*" or "home palliative*").tw. 31. (Place adj3 care) OR (Place adj3 death).tw 32. 24 or 25 or 26 or 27 or 28 or 29 or 30 or 31 33. exp *terminally ill patient/ 34. exp *terminal disease/ 35. (palliat* or terminal* or EOL or EOLC or "end of life*" or "end?of?life*" or “end of their lives” or dying or “supportive care” or “symptomatic treatment” or “irreversible condition” or “terminal condition” or fatal illness). tw 36. ((incurable* or life?limit* or advanc* or progressiv* or agressiv* or end) adj2 (diagnos* or diseas* OR illnes* or cancer* or malignan* or non-malignan* or stage* or dementia* or failure* or heart*)).tw 37. ((end or last or final) adj3 life).tw 38. (expected adj3 die).tw. 39. (imminent adj3 death).tw. 40. ("Dying soon" or "expected death" or "imminently dying" or Moribund).tw. 41. 33 or 34 or 35 or 36 or 37 or 38 or 39 or 40 42. high income country/ or exp aruba/ or exp curacao/ or exp saint martin/ or exp canada/ or exp united states/ or exp chile/ or exp uruguay/ or exp brunei darussalam/ or exp bahrain/ or exp israel/ or exp kuwait/ or exp oman/ or exp qatar/ or exp saudi arabia/ or exp united arab emirates/ or exp hong kong/ or exp macao/ or exp south korea/ or exp andorra/ or exp austria/ or exp belgium/ or exp estonia/ or exp latvia/ or exp lithuania/ or exp croatia/ or exp czech republic/ or exp hungary/ or exp poland/ or exp slovakia/ or exp slovenia/ or exp france/ or exp germany/ or exp gibraltar/ or exp united kingdom/ or exp greece/ or exp ireland/ or exp italy/ or exp liechtenstein/ or exp luxembourg/ or exp monaco/ or exp netherlands/ or exp portugal/ or exp san marino/ or exp scandinavia/ or exp spain/ or exp switzerland/ or exp vatican city state/ or exp bermuda/ or exp australia/ or exp seychelles/ or exp japan/ or exp cyprus/ or exp malta/ or exp new caledonia/ or exp guam/ or exp new zealand/ or exp taiwan/ or exp "Antigua and Barbuda"/ or exp bahamas/ or exp barbados/ or exp puerto rico/ or exp "Saint Kitts and Nevis"/ or exp "Trinidad and Tobago"/ or exp “Virgin Islands (British)”/ or exp “Virgin Islands (U.S.)”/ or exp Singapore/ or exp Channel Islands/ or exp Faroe Islands/ or exp “Isle of Man”/ or exp Northern Mariana Islands/ or exp French Polynesia/ or exp Canary Islands/ 43. 20 and 23 and 32 and 41 and 42 44. Limit 43 to humans 45. limit 44 to (yr="2004 -Current" and (adult <18 to 64 years> or aged <65+ years>)) 46. Clinical study/ 47. Case control study/ 48. Family study/ 49. Longitudinal study/ 50. Retrospective study/ 51. Prospective study/ 52. Randomized controlled trials/ 53. 51 not 52 54. Cohort analysis/ 55. (epidemiolog*or case control or cross?section* or cross section* or cohort* or Cohort analy$ or survey* or observational* or retrospectiv* or prospective* or longitudinal or routine?data or routine data or population?based or population based).tw 56. (Cohort adj (study or studies)).mp. 57. (Case control or follow up or observational or epidemiologic$ or cross sectional) adj (study or studies)).tw. 58. Exp Registration/ 59. (registr*) adj2 (cancer or death or disease or national or central or regional).tw 60. (Population*) adj2 (study* or sample* or data* or design* or based).tw 61. linked adj3 data.tw 62. administrative adj2 (data or record*).tw 63. Or/46-50,53-62 64. 45 and 63 |
| **PSYCINFO**   1. exp Geography/ 2. Health care access/ 3. Rural health/ 4. Urban health/ 5. Population/ 6. Social density/ 7. (Geograph* or topograph* or geomedicine or nosograph*).tw 8. (Rural* or urban* or remote or peri-urban or suburb* or metropolitan or non-metropolitan) adj3 (location or area)).tw 9. (City or cities or town* or village* or suburb* or county or country or countryside* or region* or provinc* or district* or state* or council* or border* or boundar* or "output area*" or constituenc* or parish* or postcode or “zip code” or distance or proximity or spatial or contextual or state-level or spatio-temporal or country-level or spatial-trend or socio-spatial or area-level or neighbo?rhood-level or location* or site* or density or nation* or sub-nation* or settlement or small-area or catchment or intra-state or inter-region*).tw 10. (CCG* or “clinical commissioning group*” or “local commissioning group*” or ICB* or “integrated care board*” or "hospital referral region*" or “'integration authorit*” or “local health board*” or “sustainability and transformation plan*” or network* or alliance* or board* or trust* or authorit*).tw 11. 1 or 2 or 3 or 4 or 5 or 6 or 7 or 8 or 9 or 10 12. (difference* or variation* or disparit* or vary or varies or distribution* or inequit* or inequalit*).tw 13. Health disparities/ 14. 12 or 13 15. Palliative Care/ 16. Hospice/ 17. Treatment duration/ 18. ((Hospice or palliat*) adj3 (care or nursing or therapy or utili?ation or "use" or usage or uptake or access* or delivery or coverage or contact or visit* or consult* or enrol?ment or admission or stay or "bed days" or "bed occupancy" or proximity or distance)).tw. 19. ((Length or duration) adj3 (Hospice or palliat*) adj3 (utili?ation or "use" or usage or uptake or access* or delivery or coverage or contact or visit* or enrol?ment or admission or stay)).tw. 20. ("Specialist palliative*" or "in?patient palliative*" or "out?patient palliative*" or "hospital palliative*" or "community palliative*" or "home palliative*").tw. 21. ((Place adj3 care) OR (Place adj3 death)).tw 22. 18 or 19 or 20 or 21 or 22 or 23 or 24 23. "death and dying"/ 24. Terminally Ill Patients/ 25. Terminal Cancer/ 26. Disease progression/ 27. (palliative or terminal* or EOL or EOLC or "end of life*" or "end?of?life*" or “end of their lives” or dying or “supportive care” or “symptomatic treatment” or “irreversible condition” or “terminal condition” or fatal illness).tw 28. ((incurable* or life?limit* or advanc* or progressiv* or agressiv* or end) adj2 (diagnos* or diseas*OR illnes* or cancer* or malignan* or non-malignan* or stage* or dementia* or failure* or heart*)). tw 29. ((end or last or final) adj3 life). Tw 30. (expected adj3 die).tw 31. (imminent adj3 death).tw. 32. (“Dying soon” or “expected death” or “imminently dying” or Moribund).tw 33. 26 or 27 or 28 or 29 or 30 or 31 or 32 or 33 or 34 or 35 34. 15 and 16 and 27 and 37 35. limit 38 to (human and "300 adulthood <age 18 yrs and older>" and yr="2004 -Current") |
| **HMIC**  **Search strategy derived using methodology by Finnegan et al. (132)**   1. (Geograph* or topograph* or geomedicine or nosograph*).mp 2. (Geograph* or topograph* or geomedicine* or nosograph*).hw 3. ((Rural* or urban* or remote or peri-urban or suburb* or metropolitan or non-metropolitan) adj3 (location or area)).mp 4. ((Rural* or urban* or remote* or peri-urban* or suburb* or metropolitan* or non-metropolitan*) and (location* or area*)).hw 5. (City or cities or town* or village* or suburb* or county or country or countryside* or region* or provinc* or district* or state* or council* or border* or boundar* or "output area*" or constituenc* or parish* or postcode or “zip code” or distance or proximity or spatial or contextual or state-level or spatio-temporal or country-level or spatial-trend or socio-spatial or area-level or neighbo?rhood-level or location* or site* or density or nation* or sub-nation* or settlement or small-area or catchment or intra-state or inter-region*).mp 6. (City* or cities* or town* or village* or suburb* or county* or country* or countryside* or region* or provinc* or district* or state* or council* or border* or boundar* or "output area*" or constituenc* or parish* or postcode* or “zip code*” or distance* or proximity* or spatial* or contextual* or state-level* or spatio-temporal* or country-level* or spatial-trend* or socio-spatial* or area-level* or neighbo?rhood-level* or location* or site* or density* or nation* or sub-nation* or settlement* or small-area* or catchment* or intra-state* or inter-region*).hw 7. (CCG* or “clinical commissioning group*” or “local commissioning group*” or ICB* or “integrated care board*” or "hospital referral region*" or “'integration authorit*” or “local health board*” or “sustainability and transformation plan*” or network* or alliance* or board* or trust* or authorit*).mp 8. (CCG* or “clinical commissioning group*” or “local commissioning group*” or ICB* or “integrated care board*” or "hospital referral region*" or “'integration authorit*” or “local health board*” or “sustainability and transformation plan*” or network* or alliance* or board* or trust* or authorit*).hw 9. 1 or 2 or 3 or 4 or 5 or 6 or 7 or 8 10. (difference* or variation* or disparit* or vary or varies or distribution* or inequit* or inequalit*).tw 11. (difference* or variation* or disparit* or vary* or varies* or distribution* or inequit* or inequalit*).hw 12. 10 or 11 13. ((Hospice or palliat*) adj3 (care or nursing or therapy or utili?ation or "use" or usage or uptake or access* or delivery or coverage or contact or visit* or consult* or enrol?ment or admission or stay or "bed days" or "bed occupancy" or proximity or distance)).tw. 14. ((Hospice* or palliat*) and (care* or nursing* or therapy* or utili?ation* or "use"* or usage* or uptake* or access* or delivery* or coverage* or contact* or visit* or consult* or enrol?ment* or admission* or stay* or "bed days*” or "bed occupancy*" or proximity or distance)).hw. 15. ((Length or duration) adj3 (Hospice or palliat*) adj3 (utili?ation or "use" or usage or uptake or access* or delivery or coverage or contact or visit* or enrol?ment or admission or stay)).tw. 16. ((Length* or duration*) and (Hospice* or palliat*) and (utili?ation* or "use"* or usage* or uptake* or access* or delivery* or coverage* or contact* or visit* or enrol?ment* or admission* or stay*)).tw. 17. (“Specialist palliative*” or “in?patient palliative*” or “out?patient palliative*” or “hospital palliative*” or “community palliative*” or “home palliative*”).tw 18. (“Specialist palliative*” or “in?patient palliative*” or “out?patient palliative*” or “hospital palliative*” or “community palliative*” or “home palliative*”).hw 19. ((Place adj3 care) OR (Place adj3 death)).tw 20. ((Place* and care*) OR (Place* and death*)).hw 21. 13 or 14 or 15 or 16 or 17 or 18 or 19 or 20 22. (palliative or terminal* or EOL or EOLC or "end of life*" or "end?of?life*" or "end of their lives" or dying or "supportive care" or "symptomatic treatment" or "irreversible condition" or "terminal condition" or fatal illness).tw. 23. (palliative* or terminal* or EOL or EOLC or "end of life*" or "end?of?life*" or "end of their lives*" or dying* or "supportive care*" or "symptomatic treatment*" or "irreversible condition*" or "terminal condition*" or fatal illness*).hw. 24. ((incurable* or life?limit* or advanc* or progressiv* or agressiv* or end) adj2 (diagnos* or diseas*OR illnes* or cancer* or malignan* or non-malignan* or stage* or dementia* or failure* or heart*)). Tw 25. ((incurable* or life?limit* or advanc* or progressiv* or agressiv* or end*) and (diagnos* or diseas* OR illnes* or cancer* or malignan* or non-malignan* or stage* or dementia* or failure* or heart*)).hw 26. ((end or last or final) adj3 life). tw 27. ((end* or last* or final*) and life*).hw 28. (expected adj3 die).tw. 29. (expected* and die*).hw. 30. (imminent adj3 death).tw. 31. (imminent* and death*).hw. 32. ("Dying soon" or "expected death" or "imminently dying" or Moribund).tw. 33. ("Dying soon*" or "expected death*" or "imminently dying*" or Moribund*).hw. 34. 24 or 25 or 26 or 27 or 28 or 29 35. 9 and 12and 23 and 30 36. limit 31 to yr="2004 -Current" |
| **CINAHL**   1. (MH "Geographic Locations+") 2. (MH "Geographic Factors+") 3. (MH "Population+") 4. (MH "Population Density") 5. (MH "Health Facilities") 6. (MH "Health Services") 7. (MH "Catchment Area (Health)") 8. (MH "Communities+") 9. (MH "Medically Underserved Area") 10. (MH "Neighborhood Characteristics+") 11. TI ( (Geograph* or topograph* or geomedicine or nosograph*) ) OR AB ( (Geograph* or topograph* or geomedicine or nosograph*) ) 12. TI ( (Rural* or urban* or remote or peri-urban or suburb* or metropolitan or non-metropolitan) n3 (location or area)) ) OR AB ( (Rural* or urban* or remote or peri-urban or suburb* or metropolitan or non-metropolitan) n3 (location or area)) ) 13. TI ( (City or cities or town* or village* or suburb* or county or country or countryside* or region* or provinc* or district* or state* or council* or border* or boundar* or "output area*" or constituenc* or parish* or postcode or “zip code” or distance or proximity or spatial or contextual or state-level or spatio-temporal or country-level or spatial-trend or socio-spatial or area-level or neighbo#rhood-level or location* or site* or density or nation* or sub-nation* or settlement or small-area or catchment or intra-state or inter-region*) ) OR AB ( (City or cities or town* or village* or suburb* or county or country or countryside* or region* or provinc* or district* or state* or council* or border* or boundar* or "output area*" or constituenc* or parish* or postcode or “zip code” or distance or proximity or spatial or contextual or state-level or spatio-temporal or country-level or spatial-trend or socio-spatial or area-level or neighbo#rhood-level or location* or site* or density or nation* or sub-nation* or settlement or small-area or catchment or intra-state or inter-region*) ) 14. TI ( (CCG* or “clinical commissioning group*” or “local commissioning group*” or ICB* or “integrated care board*” or "hospital referral region*" or “'integration authorit*” or “local health board*” or “sustainability and transformation plan*” or network* or alliance* or board* or trust* or authorit*) ) OR AB ( (CCG* or “clinical commissioning group*” or “local commissioning group*” or ICB* or “integrated care board*” or "hospital referral region*" or “'integration authorit*” or “local health board*” or “sustainability and transformation plan*” or network* or alliance* or board* or trust* or authorit*) ) 15. S1 OR S2 OR S3 OR S4 OR S5 OR S6 OR S7 OR S8 OR S9 OR S10 OR S11 OR S12 OR S13 OR S14 OR S15 16. TI ( (difference* or variation* or disparit* or vary or varies or distribution* or inequit* or inequalit*) ) OR AB ( (difference* or variation* or disparit* or vary or varies or distribution* or inequit* or inequalit*) ) 17. (MH " Palliative Care") 18. (MH " Palliative Medicine") 19. (MH " Palliative Care Nursing") 20. (MH "Hospice Care") 21. (MH "Length of Stay”) 22. (MH "Bed Occupancy”) 23. (MH “Treatment Duration”) 24. (MH “Home Health Care”) 25. (MH “Place of Death”) 26. TI ( ((Hospice or palliat*) n3 (care or nursing or therapy or utili#ation or use or usage or uptake or access* or delivery or coverage OR contact OR visit* OR consult* OR enrol#ment OR admission OR stay OR "bed days" OR "bed occupancy" OR proximity OR distance)) ) OR AB ( ((Hospice or palliat*) n3 (care or nursing or therapy or utili#ation or use or usage or uptake or access* or delivery or coverage OR contact OR visit* OR consult* OR enrol#ment OR admission OR stay OR "bed days" OR "bed occupancy" OR proximity OR distance)) ) 27. TI ( ((Length OR duration) n3 (Hospice or palliat*) n3 (utili?ation or "use" or usage or uptake or access* or delivery or coverage OR contact OR visit* OR enrol#ment OR admission OR stay)) ) OR AB ( ((Length OR duration) n3 (Hospice or palliat*) n3 (utili?ation or "use" or usage or uptake or access* or delivery or coverage OR contact OR visit* OR enrol#ment OR admission OR stay)) ) 28. Ti ( (“Specialist palliative*” or “in?patient palliative*” or “out?patient palliative*” or “hospital palliative*” or “community palliative*” or “home palliative*”) ) OR AB ( (“Specialist palliative*” or “in?patient palliative*” or “out?patient palliative*” or “hospital palliative*” or “community palliative*” or “home palliative*”) )  \| 1. TI ( (Place n3 care) OR (Place n3 death) ) OR AB ( (Place n3 care) OR (Place n3 death) ) \|  \| \| --- \| --- \|  1. S17 – S29 2. (MH "Terminally Ill Patients+") 3. TI ( (palliative or terminal* or EOL or EOLC or "end of life*" or "end?of?life*" or “end of their lives” or dying or “supportive care” or “symptomatic treatment” or “irreversible condition” or “terminal condition” or fatal illness) ) OR AB ( (palliative or terminal* or EOL or EOLC or "end of life*" or "end?of?life*" or “end of their lives” or dying or “supportive care” or “symptomatic treatment” or “irreversible condition” or “terminal condition” or fatal illness) ) 4. TI ( ((incurable* or life#limit* or advanc* or progressiv* or agressiv* or end) n2 (diagnos* or diseas*OR illnes* or cancer* or malignan* or non-malignan* or stage* or dementia* or failure* or heart*)) ) OR AB ( ((incurable* or life#limit* or advanc* or progressiv* or agressiv* or end) n2 (diagnos* or diseas*OR illnes* or cancer* or malignan* or non-malignan* or stage* or dementia* or failure* or heart*)) ) 5. TI ( ((end or last or final) n3 life) ) OR AB ( ((end or last or final) n3 life) ) 6. TI ( (expected n3 die) ) OR AB ( (expected n3 die) ) 7. TI ( (imminent n3 death) ) OR AB ( (imminent n3 death) ) 8. TI ( (“Dying soon” or “expected death” or “imminently dying” or moribund) ) OR AB ( (“Dying soon” or “expected death” or “imminently dying” or moribund) ) 9. S31 OR S32 OR S33 OR S34 OR S35 OR S36 OR S37 10. S18 and S19 and S28 and S37 Limiters - Publication Date: 20040101-20240331; Human; Age Groups: All Adult 11. (MH " Prospective studies") 12. (MH "Case control studies+") 13. (MH "Correlational studies") 14. (MH "Nonconcurrent prospective studies") 15. (MH "Cross sectional studies") 16. (MH “Registries, Disease”) 17. TI ( (epidemiolog* OR cross#section* OR cross section* OR cohort* OR survey* OR observational* OR retrospectiv* OR prospective* OR routine#data OR routine data OR population#based OR population based) ) OR AB ( (epidemiolog* OR cross#section* OR cross section* OR cohort* OR survey* OR observational* OR retrospectiv* OR prospective* OR routine#data OR routine data OR population#based OR population based) ) 18. TI ( (registr* **n2** (cancer or death or disease or national or central or regional) ) OR AB ( (registr* **n2** (cancer or death or disease or national or central or regional) ) 19. TI ( (population* n2 (study* OR sample* OR data* OR design* OR based) ) OR AB ( (population* n2 (study* OR sample* OR data* OR design* OR based) ) 20. TI ( (Linked n3 data) ) OR AB ( (Linked n3 data) ) 21. TI ( (administrative n2 (data OR record*) ) OR AB ( (administrative n2 (data OR record*) ) 22. or/45-56 23. 44 and 57 (Limiters: Publication Date: 20040101-20240331; Human; Age Groups: All Adult) |
| **SCOPUS**  ( ( TITLE-ABS ( palliative OR terminal* OR eol OR eolc OR {end of life*} OR {end-of-life*} "end of their lives" OR dying OR "supportive care" OR "symptomatic treatment" OR "irreversible condition" OR "terminal condition" OR "fatal illness" ) ) OR ( TITLE-ABS ( ( incurable* OR "life-limit*" OR advanc* OR progressiv* OR agressiv* OR end ) W/2 ( diagnos* OR diseas* OR illnes* OR cancer* OR malignan* OR non-malignan* OR stage* OR dementia* OR failure* OR heart* ) ) ) OR ( TITLE-ABS ( ( end OR last OR final ) W/3 life ) ) ) AND ( ( TITLE-ABS ( ( hospice OR palliat* ) W/3 ( utili?ation OR "use" OR usage OR uptake OR access* OR delivery OR coverage OR contact OR visit* OR consult* OR enrolment OR enrollment OR admission OR stay OR proximity OR distance OR "bed days" OR "bed occupancy" OR length OR duration ) ) ) OR ( TITLE-ABS ( ( place W/3 care ) OR ( place W/3 death ) ) ) ) AND ( ( TITLE-ABS ( geograph* OR topograph* OR geomedicine OR nosograph* ) ) OR ( TITLE-ABS ( ( rural* OR urban* OR remote OR peri-urban OR suburb* OR metropolitan OR non-metropolitan ) W/3 ( location OR area ) ) ) OR ( TITLE-ABS ( city OR cities OR town* OR village* OR suburb* OR county OR country OR countryside* OR region* OR provinc* OR district* OR state* OR council* OR border* OR boundar* OR "output area*" OR constituenc* OR parish* OR postcode OR "zip code" OR spatial OR contextual OR state-level OR spatio-temporal OR country-level OR spatial-trend OR socio-spatial OR area-level OR neighborhood-level OR neighbourhood-level OR location* OR site* OR density OR nation* OR sub-nation* OR settlement OR small-area OR catchment OR intra-state OR inter-region* ) ) OR ( TITLE-ABS ( ccg* OR {clinical commissioning group*} OR {local commissioning group*} OR icb* OR {integrated care board*} OR {hospital referral region*} OR {integration authorit*} OR {local health board*} OR {sustainability and transformation plan*} OR network* OR alliance* OR board* OR trust* OR authorit* ) ) ) AND ( TITLE-ABS ( difference* OR variation* OR disparit* OR vary OR varies OR distribution* OR inequit* OR inequalit* ) ) AND PUBYEAR > 2003 AND PUBYEAR < 2025 AND ( LIMIT-TO ( EXACTKEYWORD , "Human" ) OR LIMIT-TO ( EXACTKEYWORD , "Humans" ) OR LIMIT-TO ( EXACTKEYWORD , "Adult" ) ) |
| **Web of Science**   1. TS=(Geograph* or topograph* or geomedicine or nosograph*) 2. TS=((Rural* or urban* or remote or peri-urban or suburb* or metropolitan or non-metropolitan) near/3 (location or area)) 3. TS=((City or cities or town* or village* or suburb* or county or country or countryside* or region* or provinc* or district* or state* or council* or border* or boundar* or "output area*" or constituenc* or parish* or postcode or “zip code” or spatial or contextual or state-level or spatio-temporal or country-level or spatial-trend or socio-spatial or area-level or neighbo$rhood-level or location* or site* or density or nation* or sub-nation* or settlement or small-area or catchment or intra-state or inter-region*)) 4. TS=((CCG* or “clinical commissioning group*” or “local commissioning group*” or ICB* or “integrated care board*” or "hospital referral region*" or “integration authorit*” or “local health board*” or “sustainability and transformation plan*” or network* or alliance* or board* or trust* or authorit*)) 5. #1 OR #2 OR #3 OR #4 6. TS=((difference* or variation* or disparit* or vary or varies or distribution* or inequit* or inequalit*)) 7. TS=(((hospice or palliat*) near/3 (care or nursing or therapy or utili?ation or "use" or usage or uptake or access* or delivery or coverage or contact or visit* or consult* or enrol?ment or admission or stay or "bed days" or "bed occupancy" or proximity or distance or length or duration))) 8. TS=((“Specialist palliative*” or “in?patient palliative*” or “out?patient palliative*” or “hospital palliative*” or “community palliative*” or “home palliative*”)) 9. TS=((Place near/3 care) OR (Place near/3 death)) 10. #7 OR #8 OR #9 11. (TI=(palliative or terminal* or EOL or EOLC or "end of life*" or "end?of?life*" or “end of their lives” or dying or “supportive care” or “symptomatic treatment” or “irreversible condition” or “terminal condition” or "fatal illness")) OR (AB=(palliative or terminal* or EOL or EOLC or "end of life*" or "end?of?life*" or “end of their lives” or dying or “supportive care” or “symptomatic treatment” or “irreversible condition” or “terminal condition” or "fatal illness")) 12. (TI=((incurable* or life?limit* or advanc* or progressiv* or agressiv* or end) near/2 (diagnos* or diseas* or illnes* or cancer* or malignan* or non-malignan* or stage* or dementia* or failure* or heart*))) OR AB=((incurable* or life?limit* or advanc* or progressiv* or agressiv* or end) near/2 (diagnos* or diseas* or illnes* or cancer* or malignan* or non-malignan* or stage* or dementia* or failure* or heart*)) 13. (TI=((end or last or final) near/3 life)) OR AB=((end or last or final) near/3 life) 14. TS=((expected near/3 die)) 15. TS=((imminent near/3 death)) 16. TS=((“Dying soon” or “expected death” or “imminently dying” or moribund)) 17. #11-16 18. #16 AND #12 AND #5 AND #6 19. Limits: Timespan: 2003-12-31 to 2024-10-01 (Publication Date) |
| **Proquest and ASSIA**  TI,AB,SU(geograph* OR topograph* OR geomedicine OR nosograph*) OR TI,AB,SU((Rural* OR urban* OR remote OR peri-urban OR suburb* OR metropolitan OR non-metropolitan) NEAR/3 (location OR area)) OR TI,AB,SU(City OR cities OR town* OR village* OR suburb* OR county OR country OR countryside* OR region* OR provinc* OR district* OR state* OR council* OR border* OR boundar* OR "output area*" OR constituenc* OR parish* OR postcode OR "zip code" OR spatial OR contextual OR state-level OR spatio-temporal OR country-level OR spatial-trend OR socio-spatial OR area-level OR neighbo?rhood-level OR location* OR site* OR density OR nation* OR sub-nation* OR settlement OR small-area OR catchment OR intra-state OR inter-region*) OR TI,AB,SU(CCG* OR "clinical commissioning group*" OR "local commissioning group*" OR ICB* OR "integrated care board*" OR "hospital referral region*" OR "integration authorit*" OR "local health board*" OR "sustainability and transformation plan*" OR network* OR alliance* OR board* OR trust* OR authorit*)  AND TI,AB,SU(difference* OR variation* OR disparit* OR vary OR varies OR distribution* OR inequit* OR inequalit*)  AND TI,AB,SU((Hospice OR palliat* OR “specialist palliative*” OR “in?patient palliative*” OR “out?patient palliative*” OR “hospital palliative*” OR “community palliative*” OR “home palliative*”) NEAR/3 (utili?ation OR "use" OR usage OR uptake OR access* OR delivery OR coverage OR contact OR visit* OR consult* OR enrol?ment OR admission OR stay OR proximity OR distance OR length OR duration)) OR TI,AB,SU((Place NEAR/3 care) OR (Place NEAR/3 death))  AND TI,AB,SU(palliative OR terminal* OR EOL OR EOLC OR "end of life*" OR "end?of?life*" OR dying OR "supportive care" OR "symptomatic treatment" OR “irreversible condition” OR “terminal condition” OR "fatal illness") OR TI,AB,SU((incurable* OR life?limit* OR advanc* OR progressiv* OR agressiv* OR end) NEAR/2 (diagnos* OR diseas* OR illnes* OR cancer* OR malignan* OR non-malignan* OR stage* OR dementia* OR failure* OR heart*)) OR TI,AB,SU((end OR last OR final) NEAR/3 life)  Limits: 2004-current. |

### **Text 3: Standard data collection form**

| **Study Design and Methodology** | | | | | | | | |
| --- | --- | --- | --- | --- | --- | --- | --- | --- |
| **Study information** | **Total number of participants** | **Study Design** | **Study Aim** | **Methodology** | **Definitions** | **Inclusion criteria** | **Exclusion criteria** | **Participant characteristics** |
| Title  Authors  Study ID  Year of study  Country/countries |  |  |  | Geographic variable(s) (exposure)  Place(s) of death and/or palliative care utilisation in the last three months of life (outcome)  Statistical methods  Adjusted variables/confounders | Definition of exposure (if presented by authors)  Definition of place of death (if presented by authors) |  |  | Sociodemographic characteristics  Clinical characteristics |

| **Outcome** | **Association Adjusted?** | **Statement of direction of association** | **Other key findings** |
| --- | --- | --- | --- |
| Statistical measure of association between outcome and exposure (for example, odds ratio), with reference variable where appropriate.  Confidence intervals and p-value. | Yes/No |  |  |

### **Text 4: Newcastle-Ottawa Quality Assessment Scale (NOS)**

Note: A maximum of nine stars awarded per study in total. A study can be awarded a maximum of one star for each numbered item within the Selection and Outcome categories. A maximum of two stars can be given for Comparability in both study designs, and for Outcome in cross-sectional design.

**NOS for cohort studies**

**Selection (Maximum 4 stars)**

1) Representativeness of the exposed cohort

a) Truly representative of the average adult in the community **🟑**

b) Somewhat representative of the average adult in the community **🟑**

c) Selected group of users (for example nurses or volunteers)

d) No description of the derivation of the cohort

2) Selection of the non exposed cohort

a) Drawn from the same community as the exposed cohort **🟑**

b) Drawn from a different source

c) No description of the derivation of the non exposed cohort

3) Ascertainment of exposure

a) Secure record (for example, national database, medical records) **🟑**

b) Structured caregiver interview **🟑**

c) Written caregiver report

d) No description

4) Demonstration that outcome of interest was not present at start of study

a) Yes **🟑**

b) No

**Comparability (Maximum 2 stars)**

1) Comparability of cohorts on the basis of the design or analysis

a) Study controls for the most important factor according to its design **🟑**

b) Study controls for any additional factor **🟑**

c) Study does not control for additional factors.

**Outcome (Maximum 3 stars)**

1) Assessment of outcome

a) Independent blind assessment or reference to secure records **🟑**

b) Record linkage (for example, database records) **🟑**

c) Caregiver report

d) No description

2) Was follow-up long enough for outcomes to occur

a) Yes **🟑**

b) No

3) Adequacy of follow up of cohorts

a) Complete follow up - all subjects accounted for **🟑**

b) Subjects lost to follow up unlikely to introduce bias - small number lost - >90% follow up, or description provided of those lost) **🟑**

c) Follow up rate <90% and no description of those lost

d) No statement

**NOS for cross-sectional studies**

**Selection (Maximum 4 stars)**

1) Representativeness of the sample

a) Truly representative of the average adult in the population studied (either all subjects or random sampling) **🟑**

b) Somewhat representative of the average adult in the population studied (non-random sampling) **🟑**

c) Selected group of users (for example, nurses or volunteers) or convenience sample.

d) No description of the derivation of the sample.

2) Sample size

a) Justified and/or satisfactory according to population studied. **🟑**

b) Not justified or no information provided.

3*)* Non-response rate/data availability:

a) Satisfactory proportion of target population has data available for pre-specified outcome (for example, relevant place of death present on death certificates), or satisfactory survey response rate. **🟑**

b) Unsatisfactory proportion of target population has data available or unsatisfactory survey response rate.

4) Ascertainment of exposure

a) Secure record (for example, national database, medical records) **🟑**

b) Structured caregiver interview **🟑**

c) Written caregiver report

d) No description

**Comparability (Maximum 2 stars)**

1) Potential confounders investigated by subgroup analysis or multivariable analysis.

a) The study adjusts for the most important factor according to its design **🟑**

b) The study adjusts for any additional factor.**🟑**

c) The study does not adjust for potential confounders.

**Outcome (Maximum 3 stars)**

1) Assessment of outcome

a) independent blind assessment or reference to secure records **🟑🟑**

b) record linkage (for example, identified through database records**) 🟑🟑**

c) Caregiver report **🟑**

d) No description

2) Statistical test:

a) Statistical test used to analyse the data clearly described, appropriate, and measures of association presented including confidence intervals and P value.**🟑**

b) Statistical test not appropriate, not described, or incomplete.

### **Table 3 - Results of individual studies: Place of death**

| **Author, Title,**  **Year published, Country** | **Study design** | **Number of participants,**  **Year(s) of deaths** | **Geographic exposure(s)** | **Place(s) of death** | **Statistical analysis** | **Findings** | **Summary of direction of association** |
| --- | --- | --- | --- | --- | --- | --- | --- |
| **Assareh et al. 2019^85^**  *Variation in out-of-hospital death among palliative care inpatients across public hospitals in New South Wales, Australia.*  Australia | Cohort study | N = 25,359  Deaths between 2010-2015 | Remoteness status  Rural Versus Metropolitan hospital locality  Distance to hospital last admitted (quartiles) | Other (‘Out-of-hospital’)  versus  Hospital | Adjusted Rate Ratio  (95% CI)  Negative binomial mixed models | Remoteness status:  *REF: Major cities*   - Inner regional: 0.96 (0.82-1.13) - Outer regional and remote: 1.07 (0.91-1.25)   Distance to hospital last admitted:  *REF: 1^st^ quartile (nearest)*   - 2nd (near): 1.00 (0.89-1.14) - 3rd (far): 0.98 (0.86-1.12), - 4th (farthest): 0.95 (0.81-1.11).   Rural versus metropolitan hospital locality:  *REF: Metropolitan*   - Rural: 1.03 (0.77-1.37) | No significant findings. |
| **Assareh et al. 2020^86^**  *Variation in Hospital Use at the End of Life Among New South Wales Residents Who Died in Hospital or Soon After Discharge.*  Australia | Cohort study | N = 110,499  Deaths between 2010-2015 | Remoteness status (Major cities, Inner regional, Outer regional and remote)  Rural Versus Metropolitan hospital locality  Distance to hospital last admitted (quartiles)  Hospital palliative care unit present versus not present.  General practitioner supply (quartiles of geographically-adjusted index of relative supply)  Aged care supply (quartiles of beds per 65+ aged population) | Hospital versus  Other  (‘Out-of Hospital’) | Adjusted OR  (95% CI)  Negative binomial, log-normal, and logistic models | Remoteness status:  *REF: Major cities*   - Inner regional 1.07 (0.91-1.26), - **Outer regional 1.38 (1.12-1.70),** - **Remote 1.99 (1.33-3.01)** - Very remote 1.08 (0.54-2.25)   Rural versus metropolitan hospital locality:  *REF: Metropolitan*   - Rural 0.98 (0.79-1.22)   Distance to hospital last admitted  *REF: 1^st^ quartile (nearest)*   - **2^nd^ (Near): 1.16 (1.07-1.25)** - **3^rd^ (Far): 1.24 (1.14-1.34),** - **4^th^ (Farthest) 1.38 (1.26-1.51)**   Healthcare availability  Hospital palliative care unit:  *REF: Not present*   - Present: 0.99 (0.82, 1.19)   General practitioner supply:  *REF: 1^st^ (lowest)*   - 2^nd^ (Low): 0.95 (0.83, 1.08) - 3^rd^ (High): 0.97 (0.85, 1.10) - 4^th^ (Highest): 0.91 (0.81, 1.02)   Aged care supply:  *REF: 1^st^ (lowest)*   - 2^nd^ (Low): 0.89 (0.79-1.00), - 3^rd^ (High): 1.02 (0.87, 1.20), - 4^th^ (Highest): 1.05 (0.89, 1.23) | Hospital death more likely with outer regional or remote residence compared to major city residence  Hospital death more likely with increasing residential distance from the hospital last admitted. |
| **Cross et al. 2023^77^**  *Social Deprivation and End-of-Life Care Use Among Adults With Cancer*  United States | Cohort study | N = 33,635  Deaths between 2013-2019 | Urban  Versus  Rural | Hospital Versus  Other | Adjusted OR  (99% CI)  Multivariable logistic regression | *REF: Metropolitan*   - **Micropolitan: 0.431 (0.339-0.547)(p<0.001)** - **Small town: 0.457 (0.283-0.737)(p<0.001)** - **Rural: 0.510 (0.309-0.843)(p<0.01)** | Hospital death more likely for metropolitan residents compared to micropolitan, small town and rural residents. |
| **Gallais Serezal et al. 2016^84^** *End-of-life care for hospitalized patients with metastatic melanoma in France: a nationwide, register-based study.*  France | Cohort study | N = 3889  Deaths between 2010-2013 | Urban  Versus  Rural  Hospital facility | Hospital palliative care (inpatient unit/support bed)**^a^**  Versus  Other | Adjusted OR  (95% CI)  Multivariable logistic regression | Hospital palliative care unit versus Other:  *REF: University hospital*   - City hospital: 1.23 (0.68-2.10). - **Rural hospital: 0.33 (0.10-0.80)**   Hospital palliative care support bed**^a^** versus Other:   - **City hospital: 1.94 (1.27-2.92)** - **Rural hospital: 2.83 (1.81-4.44)** | Palliative care support bed death is more likely in rural hospitals compared to city or university hospitals. |
| **Hu et al. 2014^82^**  *Aggressiveness of end-of-life care for patients with colorectal cancer in Alberta, Canada: 2006-2009.*  Canada | Cohort study | N = 2074  Deaths between 2006-2009 | Urban Versus Rural  (according to five geographic regions) | Hospital  Versus  Other | Adjusted OR  (95% CI)  Multivariable logistic regression | *REF: Calgary (Urban/suburban)*   - **Edmonton (Urban/suburban) 1.44 (1.14-1.84),** - **North (Suburban, rural and remote) 7.71 (5.34-11.34),** - **Central (Suburban, rural, and remote) 5.65 (4.18-7.64),** - **South (Suburban, rural, and remote) 2.80 (1.97-3.99)** | Hospital deaths more likely in rural regions compared to urban. |
| **Johnson et al. 2005^78^**  *Ethnic Differences in the Place of Death of Elderly Hospice Enrollees.*  United States | Cohort study | N = 115,854  Admissions between 2000-2003 | Regions of the United States | Inpatient hospice**^b^**  Versus  Home  Inpatient hospice**^b^**  Versus  Nursing Home | Adjusted OR  (95% CI)  Multinomial logistic regression | Inpatient hospice**^b^** versus home:  *REF: South*   - Midwest: 0.95 (0.91-1.00), - **West: 0.58 (0.54-0.62),** - **Northeast: 2.00 (1.86-2.15).**   Inpatient hospice**^b^** versus nursing home:   - **Midwest: 0.49 (0.47-0.51)** - **West: 0.56 (0.52-0.60)** - **Northeast: 1.93 (1.79-2.09)** | Inpatient hospice death more likely than both home and nursing home death in the Northeast compared to West, Midwest or South. |
| **Kuo et al. 2017^69^**  *End-of-life care for head and neck cancer patients: a population-based study*  Taiwan | Cohort study | N = 25,816  Deaths between 2005-2011 | Urbanisation level  Geographic region | Hospital**^c^**  Versus  Home or hospice ward | Adjusted OR  (95% CI)  Multivariable logistic regression | Urbanisation level  *REF: Level 1 (highest)*   - Level 2: 1.07 (0.97-1.19) (p=0.149) - Level 3: 1.07 (0.96-1.20) (p=0.239) - **Level 4 (lowest): 1.23 (1.11-1.37) (p<0.001)**   Geographic region  *REF: North*   - Central: 0.92 (0.83-1.02) (p=0.113) - **South: 0.75 (0.69-0.82) (p<0.001)** - East: 0.86 (0.72-1.02) (p=0.089) | Hospital death**^c^** more likely in the most rural area or in North region (capital area) compared to home or hospice ward. |
| **Kwak et al. 2008^79^**  *Racial differences in hospice use and in-hospital death among Medicare and Medicaid dual-eligible nursing home residents.*  United States | Cohort study | N = 30,765  Deaths in between 2000-2002 | Urban  Versus  Rural | Hospital  Versus  Other | Adjusted OR  (95% CI)  Multivariate logistic regression | *REF: Rural*   - Urban: 0.94 (0.87-1.02)**^d^** | No significant findings.**^d^** |
| **Lavergne et al. 2015^83^**  *Examining palliative care program use and place of death in rural and urban contexts: a Canadian population-based study using linked data.*  Canada | Cohort study | N = 23,860  Deaths between 2003-2009 | Urban  Versus  Rural  Distance from home to palliative care programme (km) | Hospital  Versus  Other | Adjusted OR  (95% CI)  Multivariate logistic regression | Urbanicity  *REF: Rural*   - **Urban: 1.28 (1.12-1.47)(p<0.05)**   Distance to palliative care programme:  *REF: ≤10km*   - 11-50 km: 0.99 (0.92-1.06) - **>50 km: 1.52 (1.28-1.81) (p<0.05)** | Hospital death more likely if urban residence or residence >50km to a palliative care programme. |
| **Maddison et al. 2012^81^**  *Inequalities in end-of-life care for colorectal cancer patients in Nova Scotia, Canada.*  Canada | Cohort study | N = 1,201  Deaths between 2001 -2008 | Urban  Versus  Rural | Hospital  Versus  Other (‘Out-of-hospital’) | Adjusted OR  (95% CI)  Multivariate logistic regression | *REF: Rural*   - **Urban 0.48 (0.3-0.7)** | Hospital death more likely if rural residence. |
| **Morden et al. 2012^80^**  *End-of-life care for Medicare beneficiaries with cancer is highly intensive overall and varies widely.*  United States | Cohort study | N = 215,311  Deaths between 2003-2007 | Hospital bed availability | Hospital Versus Other | Adjusted Rate Ratio  Multilevel modelling | *REF: Hospital size <150 beds*   - **Hospital size 150-300 beds: 1.06 (p<0.05)** - **Hospital size >300 beds: 1.06 (p<0.05)** | Hospital death more likely for patients cared for in medium and large hospitals, compared to small hospitals. |
| **Penning et al. 2017^76^**  *"When I Said I Wanted to Die at Home I Didn't Mean a Nursing Home": Care Trajectories at the End of Life*  Canada | Cohort study | N = 11,816  Deaths between 2008-2012 | Urban  Versus  Rural | Hospital  Versus  Other  (‘Non-hospital’)  (Stratified by type of transition through the long-term care system from 2008-2012) | Adjusted OR  (CI not presented)  Multinomial logistic regression | By end-of-life care trajectory through the long-term care system:  Hospital death (from home and community centres 🡪 hospital):  *REF: Urban*   - Rural: 1.152 - Suburban: 1.039   Hospital death (from residential care/nursing home 🡪 hospital):   - **Rural: 0.174 (p<0.001)** - Suburban: 0.778 (p<0.1)   Hospital death (from home and community centres 🡪 residential care/nursing home 🡪 hospital):   - **Rural: 0.421 (p<0.001)** - Suburban: 0.975   Hospital death (from other place of care 🡪 hospital):   - **Rural: 0.369 (p<0.01)** - **Suburban: 0.605 (p<0.01)**   Non-hospital death (from home and community centres 🡪 residential care/nursing home 🡪 non-hospital):   - **Rural: 0.740 (p<0.01)** - **Suburban: 1.246 (p<0.01)**   Non-hospital death (from residential care/nursing home 🡪 non-hospital):   - **Rural: 0.408 (p<0.001)** - Suburban: 0.989   Non-hospital death (from other place of care 🡪 non-hospital):   - Rural: 0.635 (p<0.1) - Suburban: 0.865 | No clear association between hospital or non-hospital death with rural-urban residence. |
| **Phillips et al. 2023^75^**  *Inpatient Mortality in Parkinson's Disease*  United States | Cohort study | N = 710,013  Deaths/ admission from 2002-2016 | Region of United States (hospital location) | Hospital  Versus  Other | Adjusted OR  (95% CI)  Multivariate logistic regression | REF: Northeast   - **Midwest 0.78 (CI 0.74-0.83) (p<0.001)** - **South 0.84 (CI 0.79-0.88) (p<0.001)** - **West 0.82 (CI 0.78-0.87) (p<0.001)** | Hospital deaths more likely in the Northeast compared with all other US regions. |
| **Abe et al. 2022^114^**  *Municipal Characteristics of In-Home Death Among Care-Dependent Older Japanese Adults.*  Japan | Cross-sectional study | N = 544,836  Deaths in 2015 | Population density persons/100m^2^ of municipality,  Number of hospital beds, clinics and healthcare workers per 1000 people per municipality. | Home  Versus  Other | Adjusted OR  (95% CI)  Multilevel logistic regression | - Population density persons/100m^2^: 1.00 (0.96-1.05), - **Number of hospital beds per 1000 people: 0.990 (0.986-0.994),** - **Number of clinics per 1000 people: 1.13 (1.01-1.26),** - **Number of physicians per 1000 people:** - **1.04 (1.02-1.06),** - **Number of in-home service workers per 1000 people 65 years or older: 1.018 (1.005-1.031),** - Number of day service workers per 1000 people 65 years or older: 1.007 (0.996-1.018), - Number of short-stay service workers per 1000 people 65 years or older: 0.999 (0.992-1.005), - Number of long-term care facility workers per 1000 people 65 years or older: 0.995 (0.991-1.000). | Home death more likely in municipalities with higher availability of clinics, physicians, and in-home service workers per population, but with less availability of hospital beds and LTC facility workers per population. |
| **Alonso-Babarro et al. 2013^72^**  *The association between in-patient death, utilization of hospital resources and availability of palliative home care for cancer patients.*  Spain | Cross-sectional study | N=524  Deaths in 2005 | Area with palliative home care team versus  Area with no palliative home care team.**^e^** | Hospital  Versus  Other | Adjusted OR  (95% CI)  Multivariate logistic regression | - **Area with palliative home care team: 0.4 (95% CI 0.2-0.6)**   *REF: area with no palliative home care team* | Hospital death more likely in an area with no palliative care home team.**^e^** |
| **Cheon et al. 2023^105^**  *Factors associated with home death in South Korea: Using the exit data from the Korean Longitudinal Study of Aging, 2008-2018*  South Korea | Cross-sectional study | N = 1,565  Deaths in 2008, 2010, 2012, 2014, 2016, 2018 | Urban  Versus  Rural | Home  Versus  Non-home | Adjusted OR  (95% CI)  Multivariate logistic regression | - **Town/small city: 0.525 (0.371-0.744) (P<0.05)**   Large city: 0.741 (0.539-1.018) *REF: Rural area* | Home death more likely in rural areas compared to towns or small cities. |
| **Chukwusa et al. 2019^51^**  *Urban and rural differences in geographical accessibility to inpatient palliative and end-of-life (PEoLC) facilities and place of death: a national population-based study in England, UK.*  England | Cross-sectional study | N = 430,467  Deaths in 2014 | Drive time from home to nearest hospital and inpatient palliative care facility (minutes)  (Rural areas and urban areas analysed separately) | Hospice  Versus  Home  Hospital  Versus  Home | Adjusted Prevalence Ratio  (95% CI)  Modified Poisson regression | Rural  Hospice vs home:  *REF: Drive time* *0-10 mins*   - **Drive time 10-30 mins: 0.77 (0.72-0.81) (p<0.001)** - **Drive time 30-50 min 0.57 (0.52-0.62) (p<0.001)** - **Drive time over 50 min 0.42 (0.36-0.48) (p<0.001)**   Hospital vs home:   - **Drive time 10-30 mins: 0.98 (0.96-0.99) (p<0.001)** - **Drive time 30-50 mins: 0.95 (0.92-0.98) (p<0.01)** - Drive time over 50 mins: 0.79 (0.59-1.06) (p=0.11)   Urban Hospice vs home:  *REF: Drive time* *0-10 mins*   - **Drive time 10-30 mins: 0.83 (0.81-0.86) (p<0.001),** - **Drive time 30-50 mins: 0.74 (0.70-0.77) (p<0.001),** - **Drive time over 50 min 0.50 (0.43‚Äì0.59) (p<0.001)**   Hospital vs home:   - Drive time 10-30 min 0.99 (0.99-1.00) (p=0.057), - **Drive time 30-50 min 0.97 (0.95-1.00) (p<0.05),** - Drive time over 50 min 0.98 (0.86-1.13) (p=0.79) | For both rural and urban areas:  Hospice death more likely compared to home death the shorter the drive time from home to hospice.  For rural areas:  Hospital death more likely compared to home death the shorter the drive time from home to hospital. |
| **Chukwusa et al. 2020^52^**  *Regional variations in geographic access to inpatient hospices and Place of death: A Population-based study in England, UK*  England | Cross-sectional study | N = 123,088  Deaths in 2014 | Drive time to hospice in 9 government office regions (minutes) | Hospice  Versus  Home | Adjusted Proportional Ratio  (95% CIs)  Modified Poisson regression | East   - **Drive time 10-30 mins: 0.22 (0.12-0.41) (p<0.0055)** - **Drive time 30-50 mins: 0.25 (0.17-0.37) (p<0.0055)** - **Drive time >50 mins: 0.78 (0.68-0.88) (p<0.0055)**   East-Midlands   - **Drive time 10-30 mins: 0.33 (0.2-0.56) (p<0.0055)** - **Drive time 30-50 mins: 0.49 (0.39-0.62) (p<0.0055)** - **Drive time >50 mins: 0.63 (0.54-0.74) (p<0.0055)**   London   - Drive time 10-30 mins: 0.81 (0.56-1.19) - Drive time 30-50 mins or >50 mins: -   North-East   - **Drive time 10-30 mins: 0.19 (0.08-0.49) (p<0.0055)** - **Drive time 30-50 mins: 0.56 (0.37-0.85) (p<0.0055)** - Drive time >50 mins: 0.87 (0.6-1.26)   North-West   - Drive time 10-30 mins: 0.69 (0.43 - 1.09) - **Drive time 30-50 mins: 0.82 (0.7-0.96)** - **Drive time >50 mins: 0.88 (0.8-0.98)**   South-East   - Drive time 10-30 mins: 0.17 (0.01-2.65) - Drive time 30-50 mins: 0.93 (0.77-1.11) - **Drive time >50 mins: 0.8 (0.74-0.87) (p<0.0055)**   South-West   - **Drive time 10-30 mins: 0.56 (0.41-0.75) (p<0.0055)** - **Drive time 30-50 mins: 0.64 (0.54-0.77) (p<0.0055)** - Drive time >50 mins: 0.89 (0.78-1.01)   West Midlands   - Drive time 10-30 mins: - - Drive time 30-50 mins: 0.28 (0.02-5.57) - Drive time >50 mins: 0.92 (0.81-1.06)   Yorkshire and the Humber   - Drive time 10-30 mins: 0.89 (0.66-1.19) - Drive time 30-50 mins: 1.03 (0.86-1.24) - Drive time >50 mins: 1.02 (0.86-1.22) | Hospice death more likely compared to home death the shorter the drive time to a hospice in the East, East Midlands, North East, North West, South West, and West Midlands. |
| **Cohen et al. 2008^101^**  *Population-based study of dying in hospital in six European countries*  Belgium (Flanders), England, Scotland, The Netherlands, Wales, Sweden | Cross-sectional study | N= 891,780  Deaths in 2003 (2002 in Sweden). | Country | Hospital  Versus Other | Adjusted OR  (95% CI)  Multivariate logistic regression | Age 40-79:  *REF: The Netherlands*   - **Belgium (Flanders) 1.54 (1.47-1.61)** - **Sweden 4.52 (4.37-4.67)** - **Scotland 1.91 (1.84-1.97)** - **England 2.09 (2.05-2.14)** - **Wales 2.10 (2.01-2.20)**   AGE 80 and over:  *REF: The Netherlands*   - **Belgium (Flanders) 1.34 (1.28-1.40)** - **Sweden 5.17 (5.01-5.32)** - **Scotland 3.00 (2.90-3.11)** - **England 3.49 (3.41-3.57)** - **Wales 2.89 (2.77-3.02)** | For 40-79 age group, hospital death over twice as likely in England and Wales and over four times more likely in Sweden, compared to The Netherlands.  For >80 age group, hospital death was higher in Sweden, Wales, Scotland, England, and Flanders compared to the Netherlands. |
| **Forma et al. 2020^98^**  *Place of death among older people in Finland and Norway.*  Finland,  Norway | Cross-sectional study | N =  68,433  Deaths in 2011 | Urban  Versus  Rural  (Countries analysed separately) | Health centre**^g^** Versus Hospital  Nursing home Versus Hospital  ‘Not in institution’**^f^**  Versus  Hospital | Relative Risk Ratio  Multinomial regression | Finland  Health centre**^g^** vs. hospital:  *REF: Rural area*   - City 0.75, Town or suburb 0.84   Nursing home vs. hospital:   - City: 1.26, Town or suburb 0.84   Not in institution**^f^** vs. hospital:   - City 0.99, Town or suburb 0.93   Norway:  Health centre**^f^** vs. hospital N/A  Nursing home vs. hospital:  *REF: Rural area*   - **City: 0.78 (p<0.001)** - **Town or suburb: 0.83 (p<0.001)**   Not in institution**^f^** vs. hospital:   - **City: 0.60 (p<0.001)** - **Town or suburb: 0.77(p<0.001)** | In Norway, hospital death was less likely for those living in rural areas compared to cities, towns or suburbs. Rural residents were more likely to die in nursing homes or other places. |
| **Gao et al. 2014^46^**  *Geographical and temporal understanding in place of death in England (1984-2010): analysis of trends and associated factors to improve end-of-life care*  England | Cross-sectional study | N = 13,154,705  Deaths between 1984-2010 | UK Health Authority Regions | Home  Versus  Hospital  Care home  Versus  Hospital | Adjusted Proportional Ratio  (95% CI)  Multivariable modelling | **All individual variables were significant at p<0.0001.**  (Results for each region correspond to 3 time periods: 1984-92, 1993-2000, 2001-10 respectively.)  Home Versus Hospital:  *REF: North West*   - **East England 0.975 (0.969 to 0.981), 1.002 (1.001 to 1.004), 1.005 (1.004 to 1.006)** - **East Midlands 1.004 (0.997 to 1.010), 0.999 (0.998 to 1.001), 1.000 (0.999 to 1.001)** - **London 0.872 (0.867 to 0.876), 0.984 (0.982 to 0.985), 0.988 (0.987 to 0.989)** - **North East 1.016 (1.009 to 1.023), 1.000 (0.998 to 1.002), 1.000 (0.998 to 1.001)** - **South Central 1.024 (1.017 to 1.031), 1.008 (1.007 to 1.010), 1.002 (1.000 to 1.003)** - **South East Coast 1.027 (1.020 to 1.034), 1.001 (0.999 to 1.003), 1.000 (0.998 to 1.001)** - **South West 1.062 (1.056 to 1.069), 1.014 (1.012 to 1.015), 1.010 (1.008 to 1.011)** - **West Midlands 1.057 (1.051 to 1.063), 1.005 (1.004 to 1.006), 0.998 (0.997 to 0.999)** - **Yorkshire and the Humber 1.001 (0.996 to 1.007), 1.002 (1.001 to 1.004), 1.001 (1.000 to 1.002)**   Care home Versus Hospital:  *REF: North West*   - **East England 0.875 (0.844 to 0.906), 1.003 (1.002 to 1.005), 1.001 (1.000 to 1.002)** - **East Midlands 0.987 (0.954 to 1.021), 1.000 (0.998 to 1.001), 0.998 (0.997 to 1.000)** - **London 0.643 (0.623 to 0.665), 1.016 (1.015 to 1.017), 1.012 (1.011 to 1.013)** - **North East 0.931 (0.889 to 0.973), 0.992 (0.990 to 0.994), 0.994 (0.993 to 0.996)** - **South Central 0.963 (0.928 to 0.998), 1.006 (1.004 to 1.008), 1.001 (0.999 to 1.002)** - **South East Coast 0.972 (0.939 to 1.006), 0.998 (0.996 to 1.000), 0.998 (0.997 to 1.000)** - **South West 1.045 (1.013 to 1.078), 0.994 (0.992 to 0.995), 0.993 (0.992 to 0.994)** - **West Midlands 0.938 (0.908 to 0.968), 0.999 (0.997 to 1.000), 0.998 (0.996 to 0.999)** - **Yorkshire and the Humber 0.928 (0.898 to 0.959), 1.001 (1.000 to 1.003), 0.996 (0.994 to 0.997)** | Across all time periods, home death most likely in the South West and hospital death most likely in London.  Care home death was most likely in South West, and least likely in London, compared to the North West. |
| **Houttekier et al. 2010^102^**  *Place of death of older persons with dementia. A study in five European countries*  Belgium (Flanders and Brussels Capital Region),  The Netherlands,  England,  Wales,  Scotland | Cross-sectional study | N = 60,176  Deaths in 2003 | Country  Hospital beds and nursing home beds per 1,000 in health region (catchment area) | Home  Versus  Hospital  Nursing home  Versus  Hospital  Home  Versus  Nursing Home | Adjusted OR  (95% CI)  Multivariate logistic regression | Comparison of countries:  Home versus hospital:  *REF: England*   - **Belgium 7.03 (5.29-9.35)** - **The Netherlands 15.43 (12.42-19.17)** - **Scotland 1.74 (1.34-2.25)** - Wales 0.76 (0.54-1.07)   Nursing home versus hospital:   - **Belgium 4.68 (3.89-5.63)** - **The Netherlands 15.99 (13.71-18.66)** - **Scotland 1.46 (1.29-1.66)** - **Wales 1.44 (1.24-1.68)**   Home versus nursing home:   - **Belgium 2.57 (1.85-3.59)** - The Netherlands 0.80 (0.68-0.95) - **Scotland 1.47 (1.14-1.90)** - Wales 0.84 (0.57-1.23)   Healthcare input (countries pooled):  Home versus hospital:   - **Hospital beds/1,000 in health region (continuous): 0.87 (0.79-0.95)** - Nursing home beds/1,000 in health region (continuous): -   Nursing home vs hospital:   - **Hospital beds/1,000 in health region (continuous): 0.88 (0.83-0.93)** - **Nursing home beds/1,000 in health region (continuous): 1.17 (1.15-1.19)**   Home vs nursing home:   - **Hospital beds/1,000 in health region (continuous): 0.90 (0.82-0.99)** - **Nursing home beds/1,000 in health region (continuous): 0.93 (0.90-0.96)** | For patients with dementia:  Home or nursing home death compared to hospital death was more likely in The Netherlands and Belgium, compared to England.  Home death more likely than a nursing home death in Belgium and Scotland compared to England.  Hospital death most likely with increasing hospital bed availability compared to home and nursing home.  Nursing home death likelihood increases with increasing nursing home bed availability compared to home and hospital.  Home death likelihood increases with decreasing hospital or nursing home bed availability. |
| **Houttekier et al. 2010^103^**  *Place of death in metropolitan regions: Metropolitan versus non-metropolitan variation in place of death in Belgium, The Netherlands and England*  Belgium The Netherlands England | Cross-sectional study | N = 264,277  Deaths in 2003 | Metropolitan versus  Non-Metropolitan  Hospital beds and care home beds /1,000 in health region  (Countries analysed separately) | Home versus hospital  Care home versus hospital  Care home death according to healthcare availability | Adjusted OR  (95% CI)  Multivariate binomial logistic regression | Metropolitan versus Non-metropolitan  Home versus hospital (patients >44 years):  *REF: Metropolitan region*   - **Belgium non-metropolitan region 1.74 (1.58-1.91),** - **The Netherlands non-metropolitan region 1.49 (1.40-1.59),** - **England non-metropolitan region 1.26 (1.22-1.29)**   Care home versus hospital (patients >64 years):  *REF: Metropolitan region*   - Belgium non-metropolitan region: NS, - The Netherlands non-metropolitan region: NS, - **England non-metropolitan region: 1.34 (1.28-1.41)**   Healthcare availability:  Home versus hospital (patients >44 years):   - Hospital beds/1000 in health care region: NS for any country   Care home versus hospital (patients >64 years):   - Belgium Hospital beds/1000: NS - **The Netherlands Hospital beds/1000: 0.95 (0.91-0.98)** - **England Hospital beds/1000: 0.89 (0.85-0.93)** - **Belgium Care home beds/1000: 1.32 (1.24-1.41),** - **The Netherlands Care home beds/1000: 1.04 (1.02-1.05),** - **England Care home beds/1000: 1.07 (1.06-1.08)** | In Belgium, The Netherlands and England, home deaths were more likely than hospital in non-metropolitan regions compared with metropolitan regions. This disparity is the least in England.  In England, care home deaths were more likely than hospital in non-metropolitan regions compared to metropolitan regions.  In The Netherlands and England, care home death was more likely than hospital for residents with lower availability of hospital beds.  In all countries, care home death was more likely than hospital in areas with greater availability of care home beds, particularly in Belgium. |
| **Klinkenberg et al. 2005^106^**  *The last 3 months of life: care, transitions and the place of death of older people.*  The Netherlands | Cross-sectional study | N = 209  Deaths between 1995-1999 | Regions of the Netherlands:  West, North-East and South | Hospital  Versus  Home | Adjusted OR  (95% CI)**^h^**  Multivariate logistic regression | *REF = North-East*   - **West 2.16 (1.07-4.32)(p=0.03),** - **South 3.29 (1.43-7.54)(p=0.005)** | Hospital death compared to home was most likely in the South and West regions of The Netherlands compared to the North-East. (3 times and 2 times as high, respectively). |
| **Lackan et al. 2009^107^**  *Ethnic differences in in-hospital place of death among older adults in California: effects of individual and contextual characteristics and medical resource supply.*  United States | Cross-sectional study | N = 472,382  Deaths between 1999-2001 | Distance from home to hospital (miles)  Hospital beds and physicians per 1000 persons | Hospital Versus  Other | Adjusted OR  (95% CI)  Multivariate logistic regression | Distance   - **Miles from hospital: 0.992 (0.990-0.995)**   Service availability   - **Hospital beds per 1000 persons: 1.191 (1.107-1.281)** - Physicians per 1000 persons: 1.000 (0.999-1.001) | Hospital death is (slightly) more likely with shorter distance to from home to hospital, and with greater availability of hospital beds. |
| **Lee et al. 2018^110^**  *Place of death and associated gender difference in Korea 2006-2014: Evidence from exit interviews of the Korean Longitudinal Study of Ageing.*  South Korea | Cross-sectional study | N = 737  Deaths in 2008, 2010, 2012 and 2014. | Urban  Versus  Rural | Home Versus Hospital  Assisted Living Residence**^i^**  Versus  Hospital | Adjusted OR  (95% CI)  Multinomial logistic regression | Home versus hospital:  *REF: Rural*   - **Urban: 0.60 (0.42-0.87) (p<0.01)**   Assisted living residence**^i^** versus hospital:  *REF: Rural*   - Urban: 0.67 (0.38-1.18) | Home death more likely if rural resident. |
| **Lopez-Valcarcel et al. 2019^111^**  *Dying at home for terminal cancer patients: differences by level of education and municipality of residence in Spain.*  Spain | Cross-sectional study | N = 79,509  Deaths in 2015 | Region in Spain (Autonomous communities) | LTCC  Versus home/hospital   Hospital  Versus  Home/LTCC   Home  Versus  Hospital | Weighted least squares estimation coefficient (SE)  Multilevel linear probability models with weighted least squares regression | LTCC versus home/hospital:   - **Andalusia -0.0125 (0.0068) (p<0.01), Galicia -0.0248 (0.0085) (p<0.01), Catalonia 0.0995 (0.0065) (p<0.01)** Basque Country -0.0078 (0.0100), Madrid 0.0053 (0.0089), **Valencia -0.0156 (0.0069) (p<0.05), Canary Islands -0.0346 (0.0091) (p<0.01),** Dummy for capital of province or island -0.0025 (0.0124)   Hospital Versus Home/LTCC   - Andalusia -0.0178 (0.0107), Galicia -0.0058 (0.0133), Catalonia -0.0169 (0.0119), Basque Country -0.0132 (0.0157), **Madrid 0.0701 (0.0140) (p<0.01), Valencia  -0.0625 (0.0108) (p<0.01), Canary Islands 0.0852 (0.0161) (p<0.01), Dummy for capital of province or island 0.0578 (0.0194) (p<0.01)**   Home versus hospital   - **Andalusia 0.0279 (0.0099) (p<0.01), Galicia 0.0211 (0.0123) (p<0.1), Catalonia -0.0475 (0.0092) (p<0.01),** Basque Country 0.0186 (0.0145), **Madrid: -0.0781 (0.0129) (p<0.01), Valencia: 0.0759 (0.0100) (p<0.01) Canary Islands: -0.0689 (0.0150) (p<0.01), Dummy for capital of province or island: -0.582 (0.0170) (p<0.01)** | Hospital death more likely in provincial or island capital cities.  Hospital death associated with Madrid and Canary Islands.  LTCC death associated with in Catalonia.  Home death associated with Andalusia and Valencia. |
| **Luta et al. 2016^104^**  *Dying among older adults in Switzerland: Who dies in hospital, who dies in a nursing home?*  Switzerland | Cross-sectional study | N = 41,275  Deaths in 2010 | Urban  Versus  Rural  Language region (German, French, Italian)  Hospital beds (overall)/10,000 inhabitants Acute care beds per/10,000 Physicians (inpatient)/10,000 Ambulatory care (GPs & specialists)/10,000 Nursing home beds /10,000 | Hospital Versus Nursing Home | Adjusted OR  (95% CI)  Two-level logistic regression | Urbanisation  *REF: Urban*   - Peri-urban: 1.06 [1.00-1.11] - Rural: 0.95 [0.87-1.02]   Language region  *REF: German-speaking region*   - **French-speaking region: 1.43 [1.22-1.65]** - **Italian-speaking region: 1.80 [1.20-2.70]**   Healthcare availability  *REF: 1^st^ tercile (lowest)*  Hospital beds (overall)/10,000   - 2nd tercile: 0.97 [0.80-1.16] - 3rd tercile (highest): 0.95 [0.77-1.15]   Acute care beds per/10,000   - 2nd tercile: 1.10 [0.92-1.31] - 3rd tercile (highest): 1.14 [0.93-1.39]   Physicians (inpatient)/10,000   - 2nd tercile: 0.98 [0.81-1.18] - 3rd tercile (highest): 1.07 [0.85-1.33]   Ambulatory care (GPs & specialists)/10,000   - 2nd tercile: 0.84 [0.70-1.01] - **3rd tercile (highest): 0.81 [0.67-0.97]**   Nursing home beds /10,000   - **2nd tercile: 0.83 [0.70-0.98]** - **3rd tercile (highest): 0.67 [0.56-0.79)** | Hospital death more likely if in the French- or Italian-speaking regions than German-speaking region, and if lower density of ambulatory care and nursing home beds. |
| **May et al. 2020^112^**  *End-of-life experience for older adults in Ireland: results from the Irish longitudinal study on ageing (TILDA).*  Ireland | Cross-sectional study | N = 354  Deaths between 2010-2014 | Urban  Versus  Rural | Home versus hospital  Hospice (inpatient) versus hospital  Nursing home versus hospital | Adjusted Relative Risk Ratio  (95% CI)  Multinomial logistic regression | Home versus hospital:  *REF: Urban*   - Rural: 1.06 (0.62-1.82) (p=0.82)   Hospice versus hospital:   - Rural: 0.42(0.17-1.00) (p=0.05)   Nursing home versus hospital:   - **Rural: 0.33 (0.13-0.81) (p=0.02)** | Nursing home death more likely than hospital death in urban area. |
| **Menec et al. 2007^99^**  *Health care use at the end of life among older adults: Does it vary by age?*  Canada | Cross-sectional study | N=7,678  Deaths in 2000 | Urban  Versus  Rural | LTC  Versus Hospital  Home with home care Versus Hospital  Home without home care Versus  Hospital | Adjusted OR  (95% CI)  Multinomial regression | LTC versus hospital:  *REF: Urban*   - **Rural: 0.66 (0.59-0.74) (p<0.05)**   Home with home care versus hospital):   - Rural: 0.94 (0.78, 1.15)   Home, without home care versus hospital:   - Rural: 1.07 (0.91, 1.25) | LTC death compared with hospital death more likely in urban areas. |
| **Neergaard et al. 2012^108^**  *Socioeconomic position and place of death of cancer patients.*  Denmark | Cross-sectional study | N = 569  Deaths in 2006 | Urban  Versus  Rural  (by number of inhabitants in the community) | Home  Versus Other | Adjusted Prevalence Ratio  (95% CI)  Generalised linear models and Poisson regression | - 10,000-49,999 inhabitants: 0.99 (0.81 to 1.21) (p=0.918) - 50,000-99,999 inhabitants: 0.95 (0.77 to 1.17) (p=0.621) - ≥100,000 inhabitants: 0.89 (0.73 to 1.08) (p=0.234) | No significant findings. |
| **Ohlen et al. 2017^109^**  *Determinants in the place of death for people with different cancer types: a national population-based study.*  Sweden | Cross-sectional study | N = 20,710  Deaths in 2012 | Urban  Versus  Rural  Healthcare region in Sweden | Hospital versus home (living at home)  Nursing home versus home (living at home)  Hospital versus nursing home  (living in nursing home) | Adjusted OR  (95% CI)  Multivariable logistic regression | Urbanisation  *REF Rural*  Hospital vs home (living at home):   - **Urban: 1.20 (1.09-1.32)**   Nursing home vs home (living at home):   - **Urban: 1.35 (1.19-1.53)**   Hospital vs nursing home (living in a nursing home):   - Urban: 0 (interpreted as no result available)   Healthcare region  *REF: North*  Hospital vs home (living at home):   - Uppsala-Orebro: 1.03 (0.90-1.19) - **Stockholm: 1.55 (1.33-1.79)** - **Southeast: 0.60 (0.51-0.70)** - West: 0.96 (0.83-1.11) - South: 0.88 (0.76-1.01)   Nursing home vs home (living at home):   - Uppsala-Orebro: 0.90 (0.76-1.06) - **Stockholm: 0.62 (0.52-0.75)** - **Southeast: 0.80 (0.67-0.96)** - **West: 1.27 (1.07-1.50)** - **South: 0.62 (0.52-0.74)**   Hospital vs nursing home (living in a nursing home):   - Uppsala-Orebro: 1.24 (0.54-2.82) - Stockholm: 2.32 (1.00-5.41) - Southeast: 0.40 (0.12-1.33) - West: 1.30 (0.56-2.98) - South: 1.59 (0.69-3.67) | Hospital death more likely than home if urban residence (for residents living at home).  Hospital death more likely if living in the Stockholm Healthcare Region compared to the North Healthcare Region (if living at home).  Nursing home death more likely compared to home death with urban residence or living in the West region compared to the North region. |
| **Orth et al. 2021^113^**  *End-of-Life Care among Nursing Home Residents with Dementia Varies by Nursing Home and Market Characteristics.*  United States | Cross-sectional study | N = 191,435  Deaths in 2017 | Urban  Versus  Rural  Nursing home  Nursing home number of beds  Nursing home total staffing hours per resident day (10-min increments)**^j^**  Registered nurses/total staffing hours per resident day (10-min increments)  Number of hospice providers in county  Number of hospital beds per 1000 persons aged ≥65 years | Hospital  Versus  Nursing Home | Adjusted OR  (95% CI)  Multivariate logistic regression | Urbanisation  *REF: Rural Nursing Home*  Urban Nursing Home:   - Mild ADRD 1.03 [0.94-1.14] (p=0.50,) Moderate ADRD 1.05 [0.97-1.14](p=0.26), **Severe ADRD 1.17 [1.03-1.32] (p=0.015)**   Nursing home characteristics  Nursing home number of beds:   - Mild ADRD 1.00 [1.00-1.00] (p=0.33,) Moderate ADRD 1.00 [1.00-1.00] (p=.06), Severe ADRD 1.00 [1.00-1.00] (p=0.08)   Nursing home staffing hours per day (10-min increments)**^j^**:   - Mild ADRD 1.00 [0.99-1.01] (p=0.89), Moderate ADRD 1.01 [1.00-1.02] (p=0.050), Severe ADRD 1.00 [0.99, 1.02] (p=0.94)   Registered nurses/total staffing hours per resident day (10-min increments):   - Mild ADRD 0.93 [0.83-1.03] (p=0.17), Moderate ADRD 0.94 [0.86-1.03] (p=0.20), Severe ADRD 0.95 [0.83-1.09] (p=0.45)   Number of hospice providers in county:   - **Mild ADRD: 1.00 [1.00-1.00] (p<.001), Moderate ADRD 1.00 [1.00-1.00] (p<.001), Severe ADRD 1.00 [1.00-1.00] (p=0.012)**   Number of hospital beds per 1000 persons aged 65 or older:   - Mild ADRD 1.00 [1.00, 1.00] (p=0.05), **Moderate ADRD 1.00 [1.00-1.01] (p<.001)**, Severe ADRD 1.00 [1.00, 1.00] (p=0.41) | For severe ADRD, hospital death more likely if resident in urban nursing home compared to rural.  For all ADRD severities, number of hospice providers in country does not influence likelihood of death in hospital versus nursing home (OR 1.00)  For moderate ADRD, number of hospital beds does not influence hospital versus nursing home death (OR 1.00) |
| **Paredes et al. 2019^68^**  *Temporal variation in the place of death in Chile from 1997 to 2014*  Chile | Cross-sectional study | N =  1,576,392  Deaths between 1997-2014 | Urban  Versus  Rural | Home  Versus Hospital | Adjusted OR  (95% CI)  Multivariate logistic regression | Participants aged over 70 years  *REF: Urban*   - **Rural: 1.09 (1.076-1.104)** | Home death more likely if rural residence compared with hospital death. |
| **Reyniers et al. 2015^71^**  *International variation in place of death of older people who died from dementia in 14 European and non-European countries*  Belgium, the Netherlands, England, Wales, France, Italy, Spain, Czech Republic, Hungary, New Zealand, United States, Canada (Quebec excluded), and South Korea  (Mexico data excluded from this review as not high-income country) | Cross-sectional study | N = 264,604  Deaths in 2008  (2007 for United States, 2010 in Andalusia) | Urbanisation level  (Countries analysed separately)  Hospital beds/10,000 people,  LTC beds/1,000 people >65 years  GPs/100,000 people,  (Countries analysed separately)  Country**^k^** | Hospital Versus Other (For urbanization, healthcare availability)  Home versus Hospital  (For cross-national comparison)  LTC setting versus hospital  (For cross-national comparison)  LTC setting versus home (For cross-national comparison) | Adjusted OR  (95% CI)  Binomial and multinomial multivariate logistic regression | Urbanisation level: Very strong/strong  *REF: Average/weak/rural*   - Belgium NS, The Netherlands NS, **England 1.5 (1.4-1.6),** Wales NS, France 1.1 (1.0-1.2), **Italy 1.3 (1.3-1.4), Spain (Andalusia): 1.3 (1.1-1.6), Hungary 2.7 (2.2-3.3),** Czech Republic: no result, New Zealand: no result, USA: no result, **Canada: 1.2 (1.1-1.3)**, **South Korea: 1.6 (1.4-1.9).**   Healthcare availability  Hospital beds/10,000 people   - Belgium NS, The Netherlands NS, England 1.1 (1.0-1.1), Wales NS, **France 1.019 (1.015-1.024),** **Italy 1.4 (1.3-1.4)**, Spain (Andalusia) NS, Hungary: no result, Czech Republic: no result, **New Zealand 1.0 (1.0-1.1), USA 1.012 (1.009-1.014), Canada: 1.10 (1.09-1.11)**, South Korea: no result.   LTC beds/1,000 people over age 65:   - Belgium NS, The Netherlands NS, **England 0.98 (0.97-0.98),** Wales: no result, **France 0.996 (0.992-0.999), Italy 0.93 (0.92-0.94),** Spain (Andalusia) 0.99 (0.98-1.00), Hungary: no result, Czech Republic: no result, **New Zealand 1.0 (0.9-1.0), USA 0.993 (0.992-0.995),** Canada NS, South Korea: no result.   GPs/100,000 people   - Belgium 0.997 (0.994-1.0), The Netherlands NS, **England 0.96 (0.95-0.97), Wales 0.91 (0.88-0.94, France 0.976 (0.974-0.979), Italy 0.91 (0.89-0.92), Spain (Andalusia) 0.994 (0.991-0.998),** Hungary no result, Czech Republic no result, **New Zealand 1.01 (1.01-1.02), USA 0.999 (0.998-0.999), Canada: 0.96 (0.96-0.97)**, South Korea no result.   Country**^k^**  *REF: France*  Home vs Hospital:   - **Belgium: 0.5 (0.4-0.6), The Netherlands: 2.3 (1.8-2.9), England: 0.1 (0.1-0.1), Wales: 0.1 (0.1-0.1), Italy: 0.7 (0.6-0.7), Spain: 0.5 (0.4-0.5), Czech Republic: 0.3 (0.2-0.4), United States: 0.5 (0.5-0.6), Canada: 0.1 (0.1-0.1), South Korea: 0.2 (0.2-0.3)**   LTC Setting vs Hospital:   - **Belgium: 1.4 (1.2-1.5), The Netherlands: 38.0 (31.2-46.1), England: 1.4 (1.3-1.6), Wales: 1.1 (0.9-1.2), Italy: 0.5 (0.5-0.6), Spain: 0.5 (0.4-0.5), Czech Republic: 3.9 (3.1-4.7), United States: 2.6 (2.4-2.9), Canada: 1.0 (0.9-1.1), South Korea: 0.1 (0.1-0.2)**   LTC Setting vs Home:   - **Belgium: 2.7 (2.4-3.0), The Netherlands: 16.4 (14.0-19.1), England: 13.8 (12.3-15.3), Wales: 14.2 (10.8-18.8), Italy: 0.8 (0.7-0.9), Spain: 1.3 (1.1-1.4), Czech Republic: 13.2 (9.8-17.8), United States: 5.0 (4.6-5.6), Canada: 13.9 (12.1-16.0), South Korea: 0.6 (0.5-0.7)** | Hospital death more likely in strongly urbanized regions in all countries with available or significant data.  Hospital death more likely with higher hospital bed availability and lower LTC bed availability in all countries with available or significant data (New Zealand equivocal).  Hospital death more likely with fewer GPs in all countries with available or significant data, except for New Zealand where the opposite was true.  Home death most likely compared to hospital in  The Netherlands and lower in all other countries when compared with France.  LTC setting deaths most likely compared to hospital in The Netherlands (also in Belgium, England, United States and Czech Republic). Lowest in Spain, Italy and South Korea when compared to France.  LTC setting death compared to home highest in the Netherlands, England, Wales, Czech Republic, Canada, Belgium, Spain, and the United States.  LTC setting death lower in South Korea and Italy, compared with France. |
| **Sheu et al. 2019^70^**  *Hospice utilization in advanced cervical malignancies: An analysis of the National Inpatient Sample*  United States | Cross-sectional study | N = 2073  Deaths between 2007-2011. | Urban  Versus  Rural  US Region  Hospital bed size (small, medium, large) | Hospital Versus Hospice (inpatient or home care) | Adjusted OR  (95% CI)  Multivariate logistic regression | US Region  *REF: Northeast*   - Midwest 0.749 (0.53-1.01) (p=0.09) - **South 0.587 (0.45-0.77) (p=0.01)** - **West 0.496 (0.30-0.81) (p=0.01)**   Urbanisation  *REF: Urban*   - **Rural: 1.62 (1.12-2.36) (p=0.01)**   Hospital bedsize:  *REF: Large*   - Small 0.95 (CI 0.67-1.34) (p=0.91) - Medium 1.07 (0.82-1.38) (p=0.77) | Hospital death more likely than hospice (inpatient or home) if rural residence.  Hospital death more likely than hospice (inpatient or home) if in Northeast compared to South or West US regions. |
| **Temkin-Greener et al. 2012^100^**  *Rural-urban differences in end-of-life nursing home care: Facility and environmental factors.*  United States | Cross-sectional study | N =  915,688  Deaths between 2005-2007 | Urban  Versus Rural Nursing Home  (Urban, large rural, small rural, isolated rural)  Total nurse hours per resident/day No. of hospice providers in the county  Hospital beds/ 100 people aged ≥65  Distance to a hospice (per 10 mile increase). Distance to a hospital (per 10 mile increase. | Hospital Versus  Other | β coefficient  Multivariable regression | Urbanisation  *REF: Urban*   - **Large rural: 0.013 (p<0.001)** - **Small rural: 026 (p<0.001)** - **Isolated rural: 0.023 (p<0.001)**   (Difference between isolated rural and small rural not significant (p = 0.438).)  Healthcare availability   - Total nurse hours per resident per day: 0.001 (p=0.392) - No. of hospice providers in the county: 0.973x10^-4^ (p=0.124) - No. of hospital beds per 100 people aged >65: 4.911x10^-4^ (p=0.053)   Distance   - Distance to a hospice (per 10-mile increase): 0.001 (p=0.570) - **Distance to a hospital (per 10-mile increase): -0.010  (p<0.001)** | Hospital death more likely in large, small and isolated rural nursing homes compared to urban nursing homes.  Hospital death more likely with smaller distance between nursing home and hospital. |
| **Xu et al. 2020^115^**  *Assessment of changes in place of death of older adults who died from dementia In the United States, 2000–2014: a time-series cross-sectional analysis*  United States | Cross-sectional study | N = over 298,453 (presented for years 2000 and 2014 only)  Deaths between 2000-2014 | Hospital beds/1000 population  Nursing home beds/1000 older adults | Home,  Hospital,  Nursing home/LTC setting | β coefficient (SE)  Two-way fixed effects regression | Healthcare availability**^l^**  Hospital beds/1000 population   - Home: 0.04 (0.05) - Hospital: 0.06 (0.05) - NH/LTC: -0.07 (0.07)   Nursing home beds/1000 older adults   - **Home: -0.01 (0.00) (p<0.001)** - **Hospital: -0.01 (0.00) (p<0.001)** - **NH/LTC: 0.02 (0.00) (p<0.001)** | LTC death more likely, and home or hospital death less likely, with greater number of nursing home beds. |

Bold results either have a p-value <0.05 reported by study authors, or confidence intervals that exclude the null value (for example, OR 1.0).

P values are included where provided by study authors.

ADRD - Alzheimer’s disease/related dementias; CI - Confidence intervals; LTC - Long-term care facility; LTCC - Long-term care centre; NS - Not significant; OR - Odds ratio; REF - Reference category, SE - Standard Error.

a) ‘Palliative care support beds’ defined by authors as hospital beds for patients without complex symptoms justifying palliative inpatient unit admission.

b) Includes hospital-based hospice units or contract beds in area hospitals.

c) ‘Hospital’ defined by authors as acute inpatient department.

d) This result is the fully-adjusted outcome from model 2 of the study. The alternative result from model 1 was significant but not adjusted for hospice use.

e) Compares two adjacent metropolitan areas of Madrid: Alcobendas-San-Sebastian de Los Reyes and Alcala de Henares.

f) ’Not in institution’ = If the participant was not in a care facility on the day of death. For example, at home, sheltered housing, transport, outside, or had a missing place of death.

g) ’Health Centre’ is a term for a primary-care hospital. Used in Finland only.

h) This review uses the result adjusted for the most relevant covariates such as age, gender, partner status and cause of death (found in the study’s table 6, top section). We did not the use alternative result which had care arrangement as the central determinant and region as a covariate (study’s table 6, bottom section).

i) ’Assisted Living Residence’ includes nursing homes and group homes.

j) includes registered nurses, licensed practical nurses, and certified nursing assistants

k) 3/14 countries not included in this analysis (New Zealand, Hungary, Mexico) due to lack of routine death registration data available.

l) Results extracted from the two-way fixed effects model results (additional file 1).

### **Table 4 - Results of individual studies: Specialist palliative care in the last 3 months of life**

| **Author, Title,**  **Year published, Country** | **Study design** | **Number of participants,**  **Year(s) of deaths** | **Geographic exposure(s)** | **Measure of palliative care utilisation** | **Statistical analysis** | **Findings** | **Summary of direction of association** |
| --- | --- | --- | --- | --- | --- | --- | --- |
| **Receipt versus no receipt of specialist palliative care in the last 3 months of life** | | | | | | | |
| **Beccaro et al. 2007^91^**  *Inequity in the provision of and access to palliative care for cancer patients. Results from the Italian survey of the dying of cancer (ISDOC).*  Italy | Cohort study  (mortality follow-back) | N =  1271  Deaths between 2002-2003 | Region of residence | Receipt of home palliative care  (last 3 months of life) | Adjusted OR (95% CI)  Multivariate logistic regression | *REF: North West*   - North East: 1.08 (0.27-4.30) - Centre: 0.80 (0.16-3.90) - **South and islands: 0.13 (0.02-0.69)** | Receipt of specialist home palliative care more likely in the North West compared to South and Island regions of Italy. |
| **de Nooijer et al. 2020^89^**  *Primary palliative care for older people in three European countries: a mortality follow-back quality study*  Belgium, Italy, Spain | Cohort study (mortality follow-back) | N =  2329  Deaths between 2013-2014 (Belgium and Spain), 2013-2015 (Italy). | Country | Receipt of palliative care  (last 3 months of life) | Adjusted OR (95% CI)  Generalised linear mixed models | Aged 65-84  *REF: Belgium*   - **Italy: 0.56 (0.35-0.88)** - **Spain: 4.81 (2.41-9.61)**   Aged 85 or older  *REF: Belgium*   - **Italy: 0.18 (0.11-0.30)** - **Spain: 3.1 (1.71-5.53)** | Receipt of specialist palliative care more likely in Spain compared to Belgium and Italy. |
| **Elting et al. 2020^95^**  *Hospice enrollment among cancer patients in Texas covered by Medicare managed care and traditional fee-for-service plans: a statewide population-based study.*  United States | Cohort study (not defined) | N = 40,184  Deaths between 2007-2013 | Urban Versus Rural | Receipt of hospice care  (last month of life) | Adjusted OR (95% CI)  Multiple logistic regression | *REF: Rural*   - Urban: 1.008 (0.953-1.068) (p= 0.7717) | No significant findings. |
| **Ko et al. 2014^92^**  Care provided and care setting transitions in the last three months of life of cancer patients: a nationwide monitoring study in four European countries.  Belgium, the Netherlands, Italy and Spain. | Cohort study (mortality follow-back) | N=2037  Deaths between 2009-2011 | Country | Receipt of palliative care  (last 3 months of life) | Adjusted OR (95% CI)  Multivariate logistic regression | *REF: Belgium*   - **The Netherlands: 0.2 (0.2-0.3) (p<0.05)** - Italy: 1.2 (0.9-1.6) - Spain: 1.0 (0.7-1.4) | Receipt of specialist palliative care more likely in Belgium compared to the Netherlands. |
| **Mohyuddin et al. 2022^96^**  *Quality of end-of-life care in multiple myeloma: A 13-year analysis of a population-based cohort in Ontario, Canada.*  Canada | Cohort study (retrospective, population-based observational study) | N = 5,095  Deaths between 2006-2018 | Urban Versus Rural | Receipt of palliative care  (last month of life) | Adjusted OR (95% CI)  Multivariable logistic regression | *REF: Rural status 1 (1,500,000+)*   - Rural status 2 (500,000-1,499,999): 0.93 (0.73-1.19) - Rural status 3 (100,000-499,999):1.22 (0.99-1.50) - Rural status 4 (10,000-99,999): 1.04 (0.79-1.38) - Rural status 5 (<10,000):1.10(0.85-1.44) | No significant findings. |
| **Nayar et al. 2014^73^**  *Disparities in End of Life Care for Elderly Lung Cancer Patients*  United States | Cohort study (not defined) | N = 91,039  Death in 2008 | Urban Versus Rural | Receipt of hospice care (within the last 3 days of life) | Adjusted OR  Multivariable logistic regression | *REF: Metropolitan*   - **Micropolitan: 0.87 (p<0.05)** - Rural: 0.94   Remote rural: 0.91 | Receipt of hospice care in last 3 days of life more likely in metropolitan areas than micropolitan areas  (does not persist with rural areas) |
| **Orth et al. 2021^113^**  *End-of-Life Care among Nursing Home Residents with Dementia Varies by Nursing Home and Market Characteristics.*  United States | Cross-sectional study | N = 191,435  Deaths in 2017 | Urban Versus Rural Nursing home  Nursing home number of beds  Nursing home total staffing hours per resident day (10-min increments)^a^  Registered nurses/total staffing hours per resident day (10-min increments)  Number of hospice providers in county  Number of hospital beds per 1000 persons aged ≥65 years | Receipt of hospice care (last 3 months of life) | Adjusted OR  (95% CI)  Multivariate logistic regression | Urbanisation  *REF: Rural Nursing Home*  Urban Nursing Home:   - Mild ADRD 0.98 [0.90-1.08](p=0.73), **Moderate ADRD 1.08 [1.02-1.14] (p=0.006),** Severe ADRD 1.05 [0.98-1.12] (p=0.18)   Nursing home characteristics  Nursing home number of beds:   - Mild ADRD 1.00 [1.00-1.00] (p=0.31) Moderate ADRD 1.00 [1.00-1.00] (p=0.08), **Severe ADRD 1.00 [1.00-1.00] (p=0.011)**   Nursing home total staffing hours per resident day (10-min increments)^a^**:**   - Mild ADRD 1.00 [0.99-1.01] (p=0.75), Moderate ADRD 1.00 [0.99-1.00] (p=0.20), **Severe ADRD 0.99 [0.98-1.00] (p=0.032)**   Registered nurses/total staffing hours per resident day (10-min increments):   - **Mild ADRD 0.90 [0.82-1.00] (p=0.039), Moderate ADRD 0.85 [0.80-0.90] (p=<0.001), Severe ADRD 0.86 [0.80-0.93] (p<0.001)**   Number of hospice providers in county:   - **Mild ADRD 1.00 [1.00-1.00] (p=0.044),** Moderate ADRD 1.00 [1.00, 1.00] (p=0.20), Severe ADRD 1.00 [1.00-1.00] (p=0.76)   Number of hospital beds per 1000 persons aged 65 or older:   - Mild ADRD 1.00 [1.00-1.00] (p=0.39), Moderate ADRD 1.00 [1.00-1.00] (p=0.67), Severe ADRD 1.00 [1.00-1.00] (p=0.10) | For moderate ADRD - receipt of hospice care in last 3 months of life more likely in an urban nursing home compared to rural  Receipt of hospice care in last 3 months of life more likely with lower registered nurse staffing hours. |
| **Intensity or timing of specialist palliative care initiation in the last 3 months of life** | | | | | | | |
| **Hutchinson et al. 2018^93^**  *Variations in Hospice Utilization and Length of Stay for Medicare Patients With Melanoma*  United States | Cohort study (not defined) | N = 2011 (hospice users)  Deaths between 2000-2009 | Population density | Hospice length of stay (among hospice users) | Adjusted OR (95% CI)  Multivariable logistic regression | ≤3 days  *REF: Population density 250,000+*   - Population density 20,000-249,999: 0.74 (0.53-1.04) - **Population density 2500-19,999: 0.52 (0.29-0.92)** - Population density <2500: 0.79 (0.32-1.92)   ≥90 days  *REF: Population density 250,000+*   - **Population density** **20,000-249,999: 1.64 (1.18-2.28)** - Population density 2500-19,999: 1.34 (0.81-2.22)   Population density <2500: 0.68 (0.24-1.98) | Late hospice referral more likely in more densely populated areas.  Early hospice referral was more likely in less densely populated areas  (does not persist with more rural areas) |
| **Watanabe-Galloway et al. 2014^88^**  *Quality of end-of-life care among rural Medicare beneficiaries with colorectal cancer.*  United States | Cohort study (not defined) | N = 34,975  Deaths in 2008 | Urban Versus Rural | Late Hospice enrollment (<3 days before death, among hospice users) | Adjusted OR  Multivariable logistic regression | *REF: Metropolitan*   - Micropolitan 0.90 Rural 0.87 | No significant findings. |
| **Ostan et al. 2023^87^**  *End-of-life care for patients with cancer: Clinical, geographical, and sociocultural differences*  Italy | Cohort study (observational retrospective study) | N = 1,721  Deaths between 2020-2021 | Regions of Italy: Centre-North and Centre-South | Number of palliative home care services provided**^b^**  (last month of life) | Contrast estimate  General linear models | Total number of health care services by physician and nurse:  *REF: Centre-North*   - Centre-South: 1.273 (p=0.628)   Number of health care services by physician:  *REF: Centre-North*   - **Centre-South: -4.433 (p <0.001)**   Number of health care services by nurse:  *REF: Centre-North*   - **Centre-South: 5.720 (p=0.002)** | No significant difference in total palliative care activities provided across regions, however there was greater service by physicians in the Centre-North and by nurses in the Centre-South. |
| **Both receipt and intensity/timing of specialist palliative care in the last 3 months of life** | | | | | | | |
| **Kenny et al. 2024^90^**  *Specialist Palliative Care and Health Care Costs at the End of Life*  Australia | Cohort study (not defined) | N = 21,714  Deaths between 2007-2016 | Urban Versus Rural | Receipt of palliative care  (for duration ≤7 days, 7-30 days, 30-180 days) | Coefficient (SE)  Multinomial logistic regression | Participants with cancer  *REF: No use of palliative care*  Metropolitan 2:   - **≤7 days: 0.626 (0.308) (p<0.05),** 7-30 days: 0.459 (0.240), **30-180 days: -0.443 (0.187) (p<0.05)**   Metropolitan 3:   - ≤7 days: 0.511 (0.299), **7-30 days: 0.702 (0.227) (p<0.01),** 30-180 days: 0.276 (0.169)   Metropolitan 4:   - **≤7 days: 0.845 (0.309) (p<0.01), 7-30 days: 0.659 (0.245) (p<0.01),** 30-180 days: 0.036 (0.189)   Metropolitan 5:   - ≤7 days: -0.433 (0.358), **7-30 days: -0.604 (0.283) (p<0.05), 30-180 days: -1.337 (0.229) (p<0.001)**   Metropolitan 6:   - **≤7 days: 1.280 (0.300) (p<0.001), 7-30 days: 1.452 (0.227) (p<0.001), 30-180 days: 0.858 (0.169) (p<0.001)**   Regional/rural 1:   - **≤7 days: 1.315 (0.300) (p<0.001), 7-30 days:1.344 (0.232) (p<0.001), 30-180 days: 0.745 (0.177) (p<0.001)**   Regional/rural 2:   - ≤7 days: -0.471 (0.380), **7-30 days: -0.654 (0.294) (p<0.05), 30-180 days: -1.684 (0.248) (p<0.001)**   Regional/rural 3:   - **≤7 days: -0.626 (0.297) (p<0.05), 7-30 days: -0.912 (0.233) (p<0.001), 30-180 days: -1.976 (0.187) (p<0.001)**   Regional/rural 4:   - **≤7 days: -3.121 (0.642) (p<0.001), 7-30 days: -2.063 (0.339) (p<0.001), 30-180 days: -2.506 (0.258) (p<0.001)**   Regional/rural 5:   - **≤7 days: -0.882 (0.368) (p<0.05), 7-30 days: -0.598 (0.267) (p<0.05), 30-180 days: -1.628 (0.224) (p<0.001)**   Regional/rural 6:   - **≤7 days: -3.703 (1.039) (p<0.001), 7-30 days: -2.240 (0.418) (p<0.001), 30-180 days: -2.268 (0.281) (p<0.001)**   Regional/rural 7:   - **≤7 days: -1.990 (0.452) (p<0.001), 7-30 days: -1.772 (0.323) (p<0.001), 30-180 days: -2.513 (0.265) (p<0.001)**   Regional/rural 8 or 9:   - **≤7 days: -2.777 (0.571) (p<0.001), 7-30 days:  -2.181 (0.352) (p<0.001), 30-180 days: -2.125 (0.231) (p<0.001)**   Participants without cancer  *REF: No use of palliative care*  Metropolitan 2:   - ≤7 days: 0.388 (0.488), 7-30 days: 0.861 (0.841), 30-180 days: 0.004 (0.536)   Metropolitan 3:   - ≤7 days: -0.499 (0.476), **7-30 days: 1.541 (0.765) (p<0.05),** 30-180 days: 0.176 (0.477)   Metropolitan 4:   - ≤7 days: 0.340 (0.489), 7-30 days: 1.396 (0.805), 30-180 days: 0.243 (0.535)   Metropolitan 5:   - **≤7 days: -2.647 (1.092) (p<0.05),** 7-30 days:  -14.686 (806.636), 30-180 days: -1.110 (0.676)   Metropolitan 6:   - **≤7 days: 1.017 (0.445) (p<0.05), 7-30 days: 2.491 (0.769) (p<0.01), 30-180 days: 1.003 (0.463) (P<0.05)**   Regional/rural 1:   - **≤7 days: 1.067 (0.458) (p<0.05), 7-30 days: 1.748 (0.787) (p<0.05),** 30-180 days: 0.361 (0.502)   Regional/rural 2:   - ≤7 days: -0.716 (0.625), 7-30 days: -0.209 (1.037), 30-180 days: -0.512 (0.631)   Regional/rural 3:   - **≤7 days: -1.274 (0.538) (p<0.05)**, 7-30 days: 0.280 (0.804), **30-180 days: -1.512 (0.596) (p<0.05)**   Regional/rural 4:   - **≤7 days: -2.483 (0.838) (p<0.01),** 7-30 days: 0.493 (0.90), **30-180 days: -1.633 (0.740) (p<0.05)**   Regional/rural 5:   - ≤7 days: -0.837 (0.590), 7-30 days: 0.387 (0.960), **30-180 days: -1.720 (0.753) (p<0.05)**   Regional/rural 6:   - **≤7 days -2.977 (1.096) (p<0.01),** 7-30 days: -1.711 (1.262), **30-180 days: -2.655 (1.128) (p<0.05)**   Regional/rural 7:   - ≤7 days: -1.080 (0.579), 7-30 days: -0.187 (0.908), **30-180 days: -1.667 (0.843) (p<0.05)**   Regional/rural 8 or 9:   - **≤7 days: -2.999 (1.092) (p<0.01),** 7-30 days: -0.469 (0.948), **30-180 days: -1.521 (0.730) (p<0.05)** | Receipt of specialist palliative care more likely in urban areas compared to rural areas  (for any duration in participants with cancer, and for categories of 7 days or less and 31-180 days in those without cancer) |
| **Morden et al. 2012^80^**  *End-of-life care for Medicare beneficiaries with cancer is highly intensive overall and varies widely.*  United States | Cohort study | N = 215,311  Deaths between 2003-2007 | Hospital bed availability | Receipt of hospice care (last month of life)  Days in hospice (last month of life)  Hospice initiated within last 3 days of life | Adjusted rate ratio  Multilevel modelling | Hospice use, last month of life:  *REF: Hospital size <150 beds*   - **Hospital size 150-300 beds: 1.07 (p<0.05)** - **Hospital size >300 beds: 1.10 (p<0.05)**   Days in hospice, last month of life  *REF: Hospital size <150 beds*   - **Hospital size 150-300 beds: 1.02 (p<0.05)** - **Hospital size >300 beds: 1.07 (p<0.05)**   Hospice initiated, last 3 days of life  *REF: Hospital size <150 beds*   - **Hospital size 150-300 beds: 1.19 (p<0.05)** - **Hospital size >300 beds: 1.21 (p<0.05)** | Hospice use in the last month of life, number of days in hospice, and late hospice referral more likely the larger the hospital bed availability. |
| **Ramkumar et al. 2023^97^**  *Association of rurality with utilization of palliative care and hospice among Medicare beneficiaries who died from pancreatic cancer: A cohort study.*  United States | Cohort study (Retrospective cohort study) | N = 31,460  Deaths between 2016-2018 | Urban Versus Rural | Receipt of palliative care  (at ≥90 days)  Receipt of hospice care (≥3 days) | Adjusted OR (95% CI)  Logistic regression | Palliative care ≥90 days before death:  *REF: Metropolitan*   - Micropolitan 0.92 (0.78-1.09) (P=0.33) - Small town 0.91 (0.73-1.13) (p=0.40) - **Rural 1.29 (1.03-1.62) (P=0.027)**   Hospice use ≥3 days before death:  *REF: Metropolitan*   - Micropolitan 0.97 (0.89-1.05) (p=0.44) - **Small town 0.87 (0.79-0.96) (p=0.008)** - **Rural 0.86 (0.76-0.96) (p=0.010)** | Receipt of palliative care at ≥90 days before death more likely in rural compared to metropolitan areas (not significant for micropolitan and small town areas).  Receipt of hospice care ≥3 days before death more likely in metropolitan areas compared to small town and rural areas. |
| **Shepard et al. 2023^74^**  *Characteristics Associated With Mexican-American Hospice Use: Retrospective Cohort Study Using the Hispanic Established Population for the Epidemiologic Study of the Elderly (H-EPESE).*  United States | Cohort study (retrospective cohort study) | N = 1,373  Deaths between 2004-2016 | U.S. State:  Texas, California, New Mexico, Colorado, or Arizona. | Receipt of hospice care  (last month of life)  Hospice length of stay (among hospice users) | Adjusted OR (95% CI)  Multivariable logistic regression | Hospice use within last month of life  Cohort 1**^c^** (N=970):  *REF: Texas*   - California: 1.13 (0.80 - 1.60) - **New Mexico: 2.93 (1.40 - 6.17)** - Colorado: 0.77 (0.25 - 2.40) - **Arizona: 4.59 (1.10 - 19.2)**   Cohort 2**^c^** (N=403):  *REF: Texas*   - California: 1.66 (0.94 - 2.95) - New Mexico: 2.11 (0.61 - 7.28) - Colorado: 3.11 (0.25 - 39.1) - **Arizona: 6.81 (1.20 - 38.6)**   Hospice length of stay >7 days  Among hospice users (N=388):  *REF: Texas*   - California: 1.11 (0.63 - 1.96) - New Mexico: 2.66 (0.89 - 7.97) - Colorado: 1.43 (0.20 - 10.2) - Arizona: 5.23 (0.57 - 47.8) | Hospice use in the last month of life more likely if resident in Arizona or New Mexico, compared to Texas. |
| **Turkman et al. 2019^94^**  *Disparities in Hospice Utilization for Older Cancer Patients Living in the Deep South.*  United States | Cohort study (retrospective cohort study) | N = 12,725  Deaths between 2012-2015 | Hospital with palliative care physician  Hospital with inpatient palliative beds | No receipt of hospice care (last 3 months of life)  Late receipt of hospice care (≤3 days before death, among hospice users) | Unadjusted relative risk (95% CI)  Generalized log-linear models | No receipt versus receipt of hospice care (last 3 months of life)   - Board-certified palliative care physician: 0.96 (0.91-1.02) - **Inpatient palliative care beds 1.15 (1.10-1.21)**   Late receipt of hospice care (≤3 days before death)   - Board-certified palliative care physician: 0.97 (0.87-1.08) - **Inpatient palliative care beds 0.82 (0.75-0.90)** | No receipt of hospice care in the last 3 months of life more likely in hospitals with inpatient palliative care beds.  Late hospice referral more likely in hospitals without inpatient palliative care beds. |

Bold results either have a p-value <0.05 reported by study authors, or confidence intervals that exclude the null value (for example, OR 1.0).

P values are included where provided by study authors.

ADRD - Alzheimer’s disease/related dementias; CI - Confidence intervals; NS - Not significant; OR - Odds ratio; REF - Reference category, SE - Standard Error.

a) includes registered nurses, licensed practical nurses, and certified nursing assistants

b) Of patients enrolled in a national home palliative care programme. Receipt of specialist palliative care as outcome variable was assumed because all staff were trained in palliative care and activities involved liaising with general practitioners.
c) Cohort 1 = participants linked with health insurance data files. Cohort 2 = additional group with survey interview data within 2 years of death available, enabling authors to investigate variables not included in health insurance files.

**Table 5 – Key characteristics of studies reporting on rural-urban variation and hospital death (significant results only)**

| **Author, Title,**  **Year** | **Country/**  **Countries** | **Study design** | **Number of partici-pants,**  **Year(s) of deaths** | **Sociodemographic characteristics** | **Cause of death** | **Definition of rural-urban exposure** | **Definition of place(s) of death outcome** | **Summary of direction of association** |
| --- | --- | --- | --- | --- | --- | --- | --- | --- |
| **Studies associating hospital death with urban residence** | | | | | | | | |
| **Houttekier et al. 2010^103^**  *Place of death in metropolitan regions: Metropolitan versus non-metropolitan variation in place of death in Belgium, The Netherlands and England* | Belgium The Netherlands England | Cross-sectional study | N = 264,277  Deaths in 2003 | AGE >70 years: 69.5 - 74%  SEX Female: 45.5 - 51.4% | Cancer: 66 - 72.4% Heart failure:  6.4-13.8% Renal failure: 1.8 – 2.6% Liver failure:  0.2-0.4% Respiratory disease: 11.2 - 15.1% Neurodegenerative disease: 4.2 – 6.3% HIV/AIDS: 0.1-0.5% | Metropolitan: ≥400,000 inhabitants and a population density of ≥2000 inhabitants/km^2^.  Non-metropolitan: residence in the remaining parts of the country. | Home  Versus  Hospital  Care home  Versus  Hospital | In Belgium, The Netherlands and England, home deaths were more likely than hospital in non-metropolitan regions compared with metropolitan regions. This disparity is the least in England.  In England, care home deaths were more likely than hospital in non-metropolitan regions compared to metropolitan regions. |
| **Reyniers et al. 2015^71^**  *International variation in place of death of older people who died from dementia in 14 European and non-European countries* | Belgium, the Netherlands, England, Wales, France, Italy, Spain, Czech Republic, Hungary, New Zealand, United States, Canada (Quebec excluded), and South Korea  (Mexico data excluded from this review as not high-income country) | Cross-sectional study | N = 264,604  Deaths in 2008  (2007 for United States, 2010 in Andalusia) | AGE: All > 65 years  SEX Female: 61.0 - 72.9 % MARITAL STATUS Married: 23.3 – 38.3% | Dementia | No definition provided. | Hospital  Versus  Other (e.g. public space) | Hospital death more likely in strongly urbanized regions in all countries with available or significant data. |
| **Lavergne et al. 2015^83^**  *Examining palliative care program use and place of death in rural and urban contexts: a Canadian population-based study using linked data.* | Canada (Nova Scotia) | Cohort study | N = 23,860  Deaths between 2003-2009 | AGE over 65: 75.8%  SEX Female: 46.3% | Terminal illness: 43.8% Organ failure: 34.8% Frailty: 21.5% | Urban based on Census Metropolitan Area (urban area with population >100,000) or a Census Agglomeration (urban area with a population of 10,000-100,000). | Hospital  Versus  Other  (Authors could not differentiate palliative hospital beds from non-palliative beds  in examining place of death). | Hospital death more likely if urban residence. |
| **Paredes et al. 2019^68^**  *Temporal variation in the place of death in Chile from 1997 to 2014* | Chile | Cross-sectional study | N =  1,576,392  Deaths between 1997-2014 | AGE >70 years: 59.2%,  AGE (median): 74 (includes all participants aged >1 year).  SEX Female: 46.2%  MARTIAL STATUS Married: 55.2% | Not specified. | No definition provided. | Home  Versus  Hospital | Home death more likely if rural residence compared with hospital death. |
| **Forma et al. 2020^98^**  *Place of death among older people in Finland and Norway.* | Finland,  Norway | Cross-sectional study | N =  68,433  Deaths in 2011 | (For Norway)  AGE (mean): 84.9,  SEX Female: 55% | (For Norway)  Sudden death: 4.1%.  Terminal illness: 23.8%,  Organ failure: 36.9%,  Frailty: 31%  Other: 4.2% | Cities (densely populated areas), towns and suburbs (intermediate density areas) or rural areas (sparsely populated areas). | Nursing home  Versus  Hospital (secondary care hospital)  ‘Not in institution’ (not in a care facility on the day of death)  Versus  Hospital | In Norway, hospital death was less likely for those living in rural areas compared to cities, towns or suburbs. Rural residents were more likely to die in nursing homes or other places.  (Finland results not significant.) |
| **Lee et al. 2018^110^**  *Place of death and associated gender difference in Korea 2006-2014: Evidence from exit interviews of the Korean Longitudinal Study of Ageing.* | South Korea | Cross-sectional study | N = 737  Deaths in 2008, 2010, 2012 and 2014. | AGE: All >65  SEX Female: 44.9% MARITAL STATUS Married: 60.3% | Not specified. | No definition provided. | Home  Versus  Hospital | Home death more likely compared to hospital if rural residence. |
| **Ohlen et al. 2017^109^**  *Determinants in the place of death for people with different cancer types: a national population-based study.* | Sweden | Cross-sectional study | N = 20,710  Deaths in 2012 | AGE >60 years: 89.1%  SEX Female: 48.0% | Cancer  (Upper gastrointestinal 17.8%, Lower gastrointestinal 12.8%, Lung 16.3%, Breast and gynaecological 212.0%, Prostate 10.7%, Haematological 9.0%, Other 21.4%.) | Urban area defined continuous settlements or houses, with ≤200 meters between houses and with ≥200 inhabitants. | Hospital  Versus  Home  (residents living at home) | Hospital death more likely than home if urban residence (for residents living at home). |
| **Orth et al. 2021^113^**  *End-of-Life Care among Nursing Home Residents with Dementia Varies by Nursing Home and Market Characteristics.* | United States | Cross-sectional study | N = 191,435  Deaths in 2017 | AGE (mean): 85.3 - 87.8  SEX Female: 63.8 - 72.7%  MARITAL STATUS Married: 19.7 - 25.1%  ETHNICITY: White 79.8 - 86.5% | Cancer: 5.9 – 10.2% | No definition provided. | Hospital  Versus  Nursing Home  (Hospital = If discharged to hospital and died shortly after - within 8 days.) | For severe ADRD, hospital death more likely if resident in urban nursing home compared to rural nursing home. |
| **Cross et al. 2023^77^**  *Social Deprivation and End-of-Life Care Use Among Adults With Cancer* | United States (Georgia) | Cohort study | N = 33,635  Deaths between 2013-2019 | AGE <65: 65.5%  SEX Female: 46.9%  RACE: White 67.6%, Black 29.9%, Other 2.6% | Cancer (90% non-haematological) | Metropolitan >50,000 residents, micropolitan 10,000-49,000, small town 2,500-9,999, rural <2,500.  By zip code using Rural-Urban Commuting Area Codes. | Hospital  Versus  Other  (Hospital = (‘discharge  disposition was deceased’) | Hospital death more likely for metropolitan residents compared to micropolitan, small town and rural residents. |
| **Studies associating hospital death with rural residence** | | | | | | | | |
| **Assareh et al. 2020^86^**  *Variation in Hospital Use at the End of Life Among New South Wales Residents Who Died in Hospital or Soon After Discharge.* | Australia | Cohort study | N = 110,499  Deaths between 2010-2015 | AGE (median): 83 SEX Female: 48.7% Born in Australia: 76.9% | Cancer: 31.7% | Remoteness status (Major cities, Inner regional, Outer regional and remote)  By ARIA+ (Accessibility/Remoteness Index Australia Plus) of residence. | Hospital versus  Other  (‘Out-of Hospital’) | Hospital death more likely with outer regional or remote residence compared to major city residence |
| **Hu et al. 2014^82^**  *Aggressiveness of end-of-life care for patients with colorectal cancer in Alberta, Canada: 2006-2009.* | Canada | Cohort study | N = 2074  Deaths between 2006-2009 | AGE (median): 71,  SEX Female: 44.9% | Cancer  (Colon 50.5%, Rectum 49.8%, Rectosigmoid 48.1%) | Calgary and Edmonton = Urban/suburban in population size and density.  North, Central and South = mixture of suburban, rural, and remote in population size and density. | Hospital (acute care hospital)  Versus  Other | Hospital deaths more likely in rural regions compared to urban. |
| **Maddison et al. 2012^81^**  *Inequalities in end-of-life care for colorectal cancer patients in Nova Scotia, Canada.* | Canada | Cohort study | N = 1,201  Deaths between 2001 -2008 | AGE >65 years: 75.5%,  SEX Female: 46.5% | Colorectal cancer | 7 categories (urban 1-3; rural 4-7) using Statistical Area Classification and metropolitan influence zones (according to population size and urban influence). | Hospital  Versus  Other (‘out-of-hospital’ according to discharge database) | Hospital death more likely if rural residence. |
| **Menec et al. 2007^99^**  *Health care use at the end of life among older adults: Does it vary by age?* | Canada | Cross-sectional study | N=7,678  Deaths in 2000 | AGE: all >65 years, SEX female: 52.4%,  MARITAL STATUS Married: 37.0% | Circulatory  40.1%, Cancer  25.0%, Respiratory 8.7%, Digestive 4.3%, Nervous and sensory systems 4.2%, Endocrine, nutritional, and metabolic 3.9%, Genitourinary 3.0%, Injuries 3.0%, Mental disorders 2.8%, All other causes 4.9%. | No definition provided. | Long-term care facility  Versus  Hospital | Long-term care facility death compared with hospital death more likely in urban areas. |
| **May et al. 2020^112^**  *End-of-life experience for older adults in Ireland: results from the Irish longitudinal study on ageing (TILDA).* | Ireland | Cross-sectional study | N = 354  Deaths between 2010-2014 | AGE (mean): 77.7,  SEX Female: 46% | Not specified. | No definition provided. | Nursing (or residential) home  Versus  Hospital | Nursing home death more likely than hospital death in urban area. |
| **Kuo et al. 2017^69^**  *End-of-life care for head and neck cancer patients: a population-based study* | Taiwan | Cohort study | N = 25,816  Deaths between 2005-2011 | AGE (mean): 56.3, SEX female: 11.6% | Head and neck cancer | Urbanisation level: 1 (highest) to 4 (lowest). Derived using ‘population density (people/km^2^), population ratio of different educational levels, ratio of elderly people, ratio of people of working in agriculture, and number of physicians per 100,000 people’. | Hospital (acute inpatient department)  Versus  Home or hospice ward | Hospital death more likely in the most rural area compared to home or hospice ward. |
| **Sheu et al. 2019^70^**  *Hospice utilization in advanced cervical malignancies: An analysis of the National Inpatient Sample* | United States | Cross-sectional study | N = 2073  Deaths between 2007-2011. | AGE >61 years: 41.4%,  SEX: All female,  RACE: White 50.4% | Cervical cancer | No definition provided. | Hospital  Versus  Hospice (includes inpatient and home care) | Hospital death more likely than hospice (inpatient or home) if rural residence. |
| **Temkin-Greener et al. 2012^100^**  *Rural-urban differences in end-of-life nursing home care: Facility and environmental factors.* | United States | Cross-sectional study | N =  915,688  Deaths between 2005-2007 | Not provided. | Not specified. | **Urban**: city with population >50,000 and its commuting area.  **Large town**: population of 10,000 - 49,999 and its commuting area.  **Small town**: population of 2,500-9,999, some people commuting to an urban area.  **Isolated rural:** <2,500 residents, primarily commuting to an area outside an urban area.  Derived from nursing home’s zip codes using Rural-Urban Commuting Area Codes. | Hospital  Versus  Other | Hospital death more likely than other places of death in large, small and isolated rural nursing homes compared to urban nursing homes. |
